# Kiss and spit metabolomics highlight the role of host purine metabolism during pathogen infection

**DOI:** 10.1128/msphere.00256-26

**Published:** 2026-06-15

**Authors:** Gina M. Gallego-Lopez, William J. Olson, Andres M. Tibabuzo-Perdomo, David Stevenson, Daniel Amador-Noguez, Grant Lawrence, James W. Leahy, Emmanuel Contreras Guzman, Melissa C. Skala, Laura J. Knoll

**Affiliations:** 1 Morgridge Institute for Research 145254, Madison, Wisconsin, USA; 2 Department of Medical Microbiology and Immunology, University of Wisconsin 732057, Madison, Wisconsin, USA; 3 HHMI, University of Wisconsin 5228, Madison, Wisconsin, USA; 4 Department of Bacteriology, University of Wisconsin 205263, Madison, Wisconsin, USA; 5 Department of Chemistry, University of South Florida 429154, Tampa, Florida, USA; 6 The Florida Center for Excellence for Drug Discovery and Innovation, University of South Florida 7831, Tampa, Florida, USA; 7 Department of Molecular Medicine, Morsani College of Medicine, University of South Florida 685045, Tampa, Florida, USA; 8 Department of Biomedical Engineering, University of Wisconsin 5228, Madison, Wisconsin, USA; Institut Pasteur de Montevideo, Montevideo, Uruguay

**Keywords:** *Toxoplasma gondii*, host–pathogen interaction, cN-II enzyme, metabolomics, purines

## Abstract

Intracellular bacteria and protists rely on the host cell to supply many metabolites, but the mechanisms through which pathogens manipulate host metabolism to their benefit are not understood. Here, we demonstrate that when the obligate intracellular parasite *Toxoplasma gondii* secretes its rhoptry organelle contents into the host cytoplasm before invasion—a process called “kiss and spit”—host cell metabolite abundance is altered in nucleotide synthesis, the pentose phosphate pathway, glycolysis, and amino acid synthesis. U-13C6-labeling metabolomics confirmed that kiss and spit increased the flow of carbon through the pentose phosphate pathway and nucleotide synthesis. An increase in 2,3-bisphosphoglycerate abundance led us to investigate the activation of host cytosolic nucleosidase II (cN-II) to provide purines for the parasite. We found that *T. gondii* manipulates the host cN-II enzyme to dephosphorylate GMP and IMP that it needs for replication. Furthermore, we found that the approved anti-cancer drug fludarabine, which inhibits cN-II, also inhibits *Toxoplasma* replication. These results reveal *Toxoplasma* host cell manipulation and highlight potential therapies for toxoplasmosis.

**IMPORTANCE:**

A fundamental challenge in parasitology is understanding how intracellular parasites rapidly reprogram host metabolism to support replication. This study reveals that *Toxoplasma gondii* initiates profound metabolic reprogramming through a “kiss-and-spit” mechanism, secreting effector molecules without invasion. We demonstrate that *T. gondii* specifically hijacks host cytosolic 5′-nucleotidase II (cN-II) by elevating 2,3-bisphosphoglycerate levels, which allosterically activates this enzyme to generate purines essential for parasite survival. Genetic deletion of host cN-II significantly impairs parasite replication, establishing cN-II as a critical host dependency factor. These findings have important implications for antiparasitic drug development while advancing our understanding of purine metabolism in apicomplexan parasites. More broadly, elucidating the molecular mechanism linking parasite effector secretion to specific host enzyme activation provides a framework for understanding metabolic manipulation across other intracellular pathogens.

## INTRODUCTION

Intracellular pathogens need a suitable host cell to replicate and provide a nutrient supply. Most of these host cells are not highly metabolically active, so these microorganisms need to reprogram their host cells to support microbial replication. For example, the obligate intracellular parasite *Toxoplasma gondii* relies on the host cell to provide essential metabolites such as arginine, tyrosine, tryptophan, purines, cholesterol, or sphingolipids (1–11). These auxotrophies present an opportunity for host-directed therapies to inhibit pathways that *T. gondii* requires, but a healthy uninfected host cell does not. Current therapeutics for *T. gondii* treatment are limited, must be given in combinations, and all target parasite metabolism (12).

To develop a new generation of therapies that limit the growth of *T. gondii,* we must first characterize how the parasite changes host metabolism. Direct quantification of the host metabolome during infection is complicated by the technical challenge of separating *T. gondii* and host cell metabolites. Studies have used genetically manipulated host cells to identify essential host pathways for *T. gondii* growth, including arginine synthesis and cholesterol scavenging (1, 12, 13). Other work has used gene expression analysis and proteomics to show that *T. gondii* changes the transcription and translation of enzymes in multiple host pathways, including the pentose phosphate pathway (PPP), glycolysis, and nucleotide synthesis (14, 15).

*T. gondii* is incapable of synthesizing purines and must import them from their host (16–18). To incorporate purines into the parasite, a series of events are required. First, purines must be fully dephosphorylated before they can be taken up and used by the parasite (7, 19). Purine nucleotide levels are regulated by host nucleotidases that hydrolyze these nucleotides into nucleosides (20), which are then transported in membrane transporters to the interior of the parasite (21, 22). These host 5′-nucleotidases dephosphorylate non-cyclic nucleoside monophosphates to nucleosides and inorganic phosphate. Eight human 5′-nucleotidases with different subcellular localization have been identified (Table S1): ecto-5′-nucleotidase, cytosolic 5′-nucleotidase IA, cytosolic 5′-nucleotidase IB (cN-1B), cytosolic 5′-nucleotidase II (cN-II), cytosolic 5′-nucleotidase IIIA (cN-3A), cytosolic 5′-nucleotidase IIIB (cN-3B), cytosolic deoxynucleotidase, and mitochondrial deoxynucleotidase (23–37). Most are localized inside the cell cytoplasm, some in the nucleus, in exosomes, or in the mitochondria, and one is bound to the plasma membrane. Among them, cN-II catalyzes both the hydrolysis of several nucleoside monophosphates and the phosphate transfer from a nucleoside monophosphate donor to the 5′ position of a nucleoside acceptor (26, 27, 29). cN-II acts on substrates such as IMP, GMP, and their corresponding deoxy-derivatives (29), and has been reported in many human and vertebrate tissues. Its activity is high in cells with elevated DNA synthesis as well as in cancerous tissue compared to its normal parental tissue, making it a target for cancer chemotherapeutics (29).

**Fig 1:**
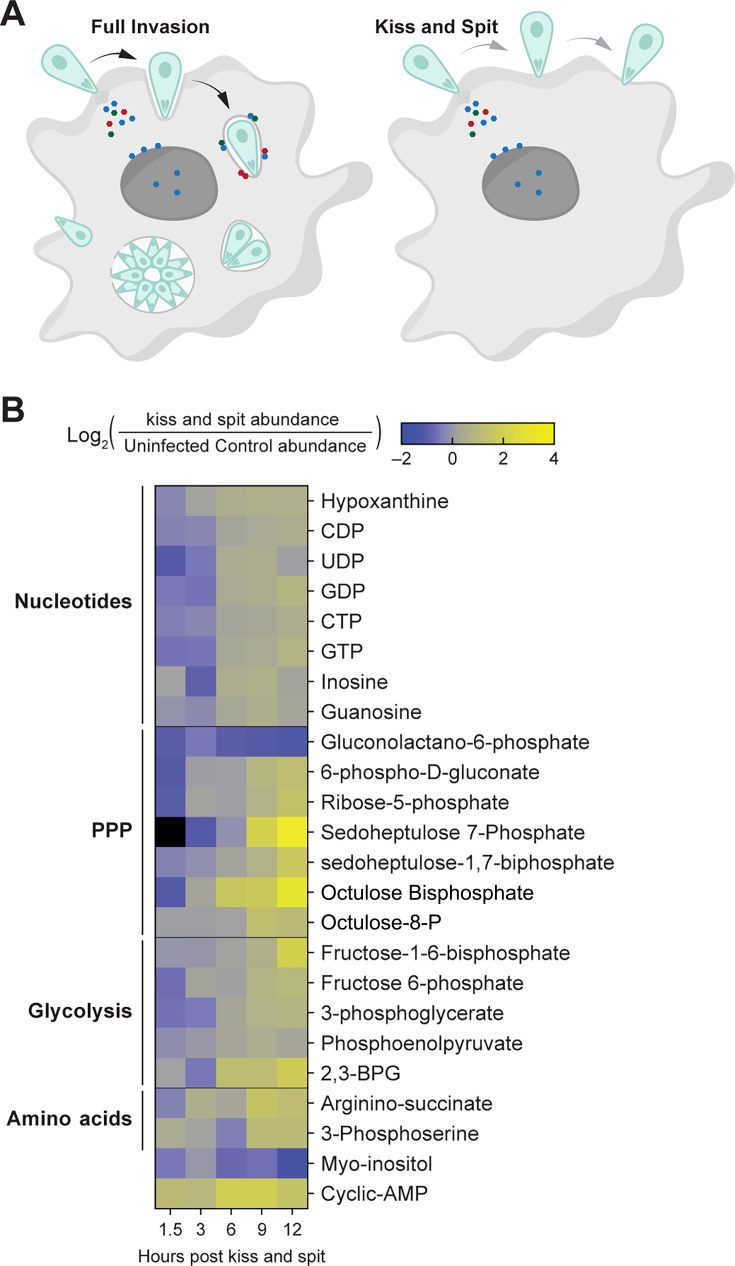
Kiss and spit. (**A**) Schematic representation of full invasion and kiss and spit. Kiss and spit is the pre-invasion process in which the contents of the parasite rhoptry organelles are secreted into the host cytoplasm. It was studied using the actin inhibitor cytochalasin D, which allows the parasite to attach but not invade the host cell. Some rhoptry proteins are directed to the nucleus of the host cell, others form the parasitophorous vacuoles (PV), and others are believed to be secreted into the host cytoplasm. (**B**) ME49-*T. gondii* kiss and spit remodels host metabolism. Heatmap shows selected metabolite relative abundance in human cells over 12 hours after *T*. gondii kiss and spit. Triplicate kiss and spit-treated and untreated dishes of human foreskin fibroblasts (HFFs) were metabolically quenched (*N* = 3), and metabolites were extracted at five time points (1.5, 3, 6, 9, and 12 HPK&S). Metabolites were quantified using HPLC-MS, and metabolites were identified with known standards. Kiss and spit-treated sample abundances were averaged and normalized to the average control abundance, then log base 2 transformed [log_2_ (kiss and spit abundance / average control abundance)] with blue being less abundant and yellow more abundant. Metabolites were selected for inclusion based on whether they could be confidently identified, and their abundance changed during infection. Metabolites were classified as nucleotides, PPP, glycolysis, and amino acids. Values represented in this heatmap and their *P*-value (calculated by two-way analysis of variance [ANOVA] adjusted by Tukey’s multiple comparisons) are in Table S2A; the complete metabolomics analyses are in Fig. S1A; and the global analyses are in Fig. S1B.

cN-II activity is modulated by effector molecules such as 2,3-bisphosphoglycerate (2,3-BPG), ATP, and GTP in an allosteric site (29, 38). In our prior metabolomic studies of *T. gondii-*infected host cells, we saw an increased abundance of 2,3-BPG (39). In the present research study, we have found that *T. gondii* kiss and spit generates high levels of 2,3-BPG, similar to full infection. Kiss and spit is a pre-invasion process in which the *T. gondii* rhoptry organelles are secreted into the host cytoplasm (40, 41) (Fig. 1A). The rhoptries contain an estimated 50 proteins and lipids, most of which are functionally uncharacterized, and the impact of kiss and spit on cellular metabolites is unknown (40, 42–45). The parasite uses effector molecules from the rhoptries or dense granules to activate biochemical mechanisms of manipulation of host enzymes and host transcriptional factors (46). Cytochalasin D (Cyt D) (47, 48) or mycalolide B (49–52) function as actin polymerization inhibitors that prevent invasion, allowing the host changes associated with kiss and spit to be studied independently of parasite invasion and replication. In this study, we determined how *T. gondii* rhoptry contents discharged during kiss and spit remodel the host metabolism using mass spectrometry-based metabolomics. We suggest that the high levels of 2,3-BPG generated by kiss and spit act as an allosteric regulator of the cN-II enzyme to upregulate its activity, which in turn generates purine nucleosides for *T. gondii*. We also evaluated the FDA-approved cN-II inhibitor, fludarabine, to block *T. gondii* replication and purine abundance.

## RESULTS

### *T. gondii* kiss and spit selectively remodels host cell metabolism

We performed a time course mass spectrometry-based analysis of the impact of *T. gondii* on host cell metabolism using a kiss and spit cell culture model and a metabolomics protocol that has demonstrated effectiveness specifically for the metabolites of interest in mammalian cell cultures (39, 53–56). By pretreating *T. gondii* with the actin polymerization inhibitor Cyt D, we allowed *T. gondii* to secrete the contents of the rhoptries into host cells while preventing infection (48). This metabolic system is simplified compared to the full infection model, containing only host metabolism and a discrete pool of parasite rhoptry factors. Our analysis found that the parasite rhoptry contents changed the host metabolism in nucleotide synthesis, the PPP, glycolysis, tricarboxylic acid cycle (TCA), amino acid synthesis, and the abundance of the signaling molecules myo-inositol and cyclic AMP (cAMP) (Fig. 1B; Fig. S1A and B).

Several metabolic pathways were altered by kiss and spit (Fig. 1; Fig. S1; Table S2). In the PPP, gluconolactone-6-phosphate was depleted in host cells at all time points denoted as hours post kiss and spit (HPK&S). In contrast, 6-phosphogluconate, the nucleotide precursor ribose-5-phosphate, sedoheptulose-7-phosphate (S7P), sedoheptulose-1,7-bisphosphate (SBP), octulose-1,8-bisphosphate (OBP), and octulose 8-phosphate (O8P) were significantly more abundant at 9 and 12 HPK&S. The increase in abundance of SBP, S7P, OBP, and O8P shares a similar pattern to the *T. gondii* full infection metabolome, although the host does not possess the sedoheptulose biphosphatase enzyme, present in *T. gondii,* that converts SBP to S7P (39).

Other metabolic pathways altered by kiss and spit include multiple glycolytic intermediates that were significantly more abundant at 9 and 12 HPK&S, including fructose-6-phosphate, fructose-1,6-bisphosphate, 3-phosphoglycerate, phosphoenolpyruvate, and 2,3-BPG. Two amino acid precursors, 3-phosphoserine and argininosuccinate, significantly increased in abundance by kiss and spit. 3-Phosphoserine is the final metabolite in the serine biosynthetic pathway (57) and was more abundant at 9 and 12 HPK&S. Similarly, argininosuccinate is the final intermediate in the arginine synthesis pathway, and it was more abundant at 9 and 12 HPK&S (58). Kiss and spit also altered the abundance of two metabolites that act as signaling molecules: myo-inositol was depleted throughout the time course, while cAMP was more abundant after kiss and spit at 6 and 9 HPK&S. Finally, several nucleotide metabolites increased in abundance after kiss and spit, including multiple phosphorylated forms of purines and pyrimidines. CDP, UDP, GDP, GTP, and CTP were statistically significantly more abundant between 6 and 12 HPK&S, as was the precursor metabolite hypoxanthine.

To ensure that our findings were due to kiss and spit, we examined host metabolism after treatment with heat-killed parasites, *T. gondii* infection-conditioned media, and a control of parasite plus Cyt D. The three controls showed different metabolomic profiles than kiss and spit (Fig. S1C). Thus, host cells were treated with heat-killed parasites or a heated media negative control for 12 hours before their metabolites were analyzed. The results indicate that the presence of dead parasites was not causing the same metabolic shift in host cells induced by kiss and spit (Fig. S2A; Table S2). Second, conditioned media was taken from heavily infected cells and swapped onto uninfected dishes for 12 hours before metabolites were analyzed, using media from uninfected cells as a negative control. The changes induced by conditioned media were significantly different from those induced by kiss and spit (Fig. S2B; Table S2), indicating that factors secreted into the media during infection were not responsible for the changes observed after kiss and spit. Third, to ensure that the changes we observed in host metabolism were not attributable to *T. gondii* remaining in the dish, we performed a control to measure the metabolic contribution of the parasites. Parasites were incubated with Cyt D for 12 hours before extracting metabolites. Media with Cyt D but no parasites was used as a control. Different parasite metabolites were detected (Fig. S2C; Table S2), but they did not overlap with the metabolites detected in kiss and spit host cells (Fig. 1), indicating that the contribution of the parasites in the dish to our metabolic data is negligible, and the changes we observed were occurring within the host.

### U-13C6-glucose labeling during *T. gondii* infection highlights differences in kiss and spit and full infection

**Fig 2:**
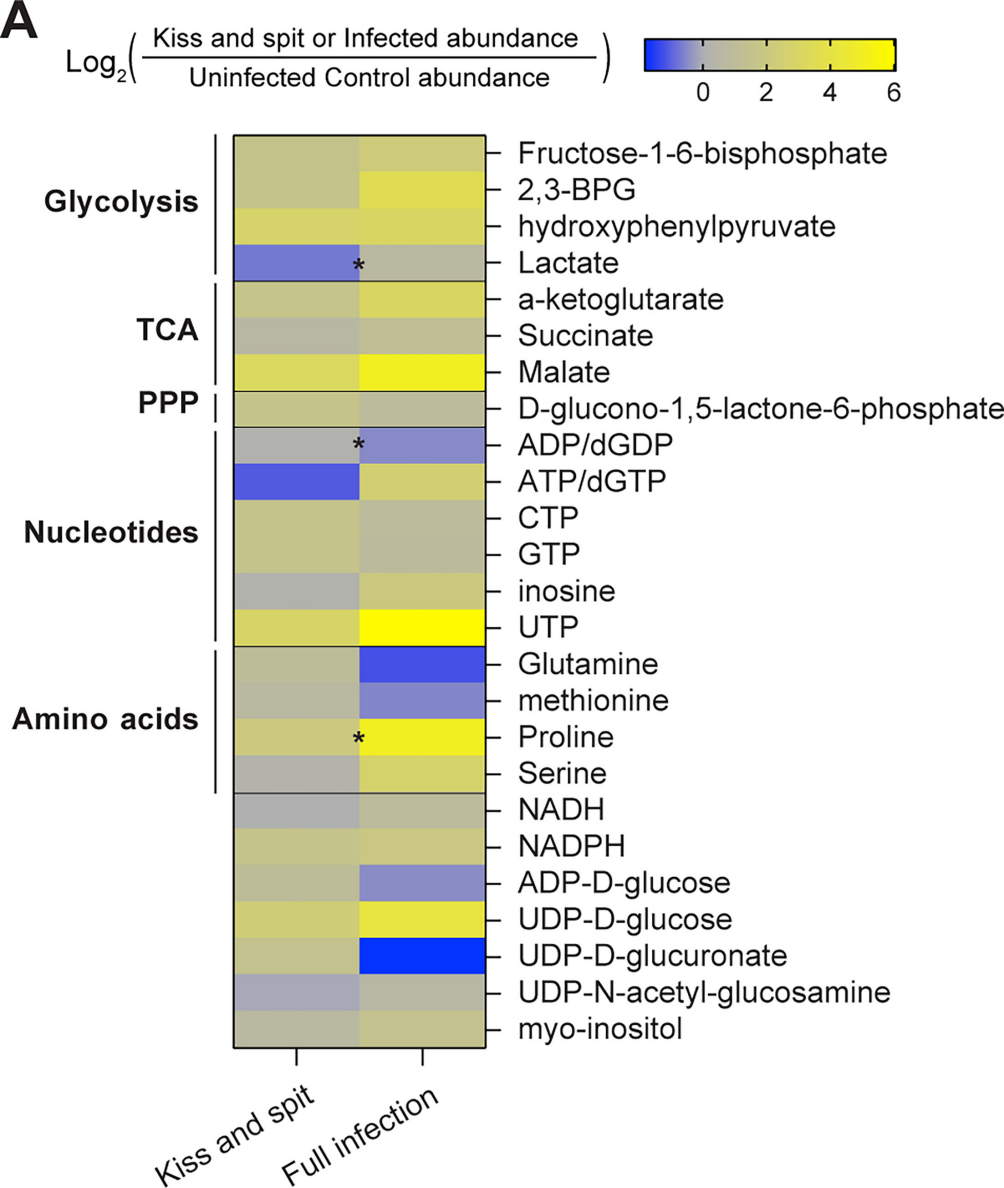
Glucose labeling during *T. gondii* infection and kiss and spit. (A) Heatmap of U-13C6-glucose-labeled metabolites in ME49 *T. gondii* kiss and spit and full infected HFF cells at 9 HPK&S and HPI. Scales show the log_2_ fold change in percentage of U-13C6-glucose labeling with respect to the uninfected control, with blue being less abundant and yellow more abundant. U-13C6-glucose labeling was performed for 30 minutes after 9 HPI in kiss and spit and full infected HFF cells; data were corrected for natural abundance. Kiss and spit or full infection percentage of labeling were normalized to the average of their respective control labeling percentage then log base 2 transformed [log_2_ (kiss and spit percentage of labeled/average of uninfected control + CD percentage of labeling)] or [log_2_ (full infection percentage of labeled / average of uninfected control percentage of labeling)]. Metabolites were selected for inclusion based on whether they could be confidently identified, and their labeling was constant in kiss and spit and in full infection. Metabolites were classified in nucleotides, PPP, glycolysis, TCA, and amino acids. * represents statistical significance comparing kiss and spit vs full infection. Alpha = 0.05; 0.1234 (ns), 0.0332 (*), 0.0021 (**), 0.0002 (***), <0.0001 (****). Values represented in this heatmap and their *P*-values (calculated by multiple *t*-test) are in Table S2B; the complete metabolomics analyses are in Fig. S3. *N* = 7.

To confirm kiss and spit results, we performed U-13C6-glucose labeling metabolomics for 30 minutes at 9 HPK&S, or hours post-infection (HPI). The major dynamic changes were observed in glycolysis, TCA, some nucleotides, and amino acids (Fig. 2). Lactate, ADP, and proline showed significant differences between kiss and spit and full infection at 9 HPI (Table S2). When analyzing the mass isotopolog distribution, kiss and spit compared to full infection showed significantly higher labeling of D-erythose-4-phosphate (M+3), ADP-glucose (M+6), and ADP (M+1 and M+2) (Fig. S3). Kiss and spit in comparison to full infection showed significantly lower labeling of ATP (M+5 and M+6), O-acetyl L-serine (M+2), proline (M+2), α-ketoglutarate (M+2), malate (M+2), and glutamate (M+2), suggesting more TCA cycle and amino acid synthesis during full infection (Table S2). Additionally, the labeling of lactate and FBP (M+1, M+2, and M+3) (59) suggests large carbon contribution by gluconeogenesis in both kiss and spit and full infection. A schematic representation based on our results and literature shows the flow from glycolysis toward PPP, nucleotides, and nucleotide sugars (Fig. S3B) (60–74).

It is important to highlight the label of phenylalanine (Phe) in kiss and spit cells (Fig. S3A) and the presence of this amino acid in heat killed parasites and parasite plus Cyt D controls (Fig. S2A and C), full infected cells (Fig. S4), and not present in uninfected cells or conditioned media control (Fig. S2B). Human cells are Phe auxotrophs, as it is absent in mammals, which makes it a promising therapeutic anti-parasitic target (75–77). Thus, the Phe observed in our metabolomics came from the parasite and not from the host, as it has been identified previously (78). *T. gondii* can synthesize Phe through the shikimate pathway given an adequate supply of a specific precursor, phenylpyruvate (79, 80). The shikimate pathway is essential for survival of the apicomplexan parasites such as *Plasmodium falciparum*, *T. gondii*, and *Cryptosporidium parvum*. Intracellular *T. gondii* tachyzoites rapidly uptake Phe from the host cells and not from the media (79). Despite the capacity for *de novo* synthesis, *T. gondii* still scavenges Phe and other amino acids, which are essential for the host (1). *T. gondii* infection induces host lysosomal amino acid accumulation, including Phe, potentially available for the parasite’s use (78).

### *T. gondii* infection and kiss and spit have conserved shifts in nucleotide metabolism

**Fig 3:**
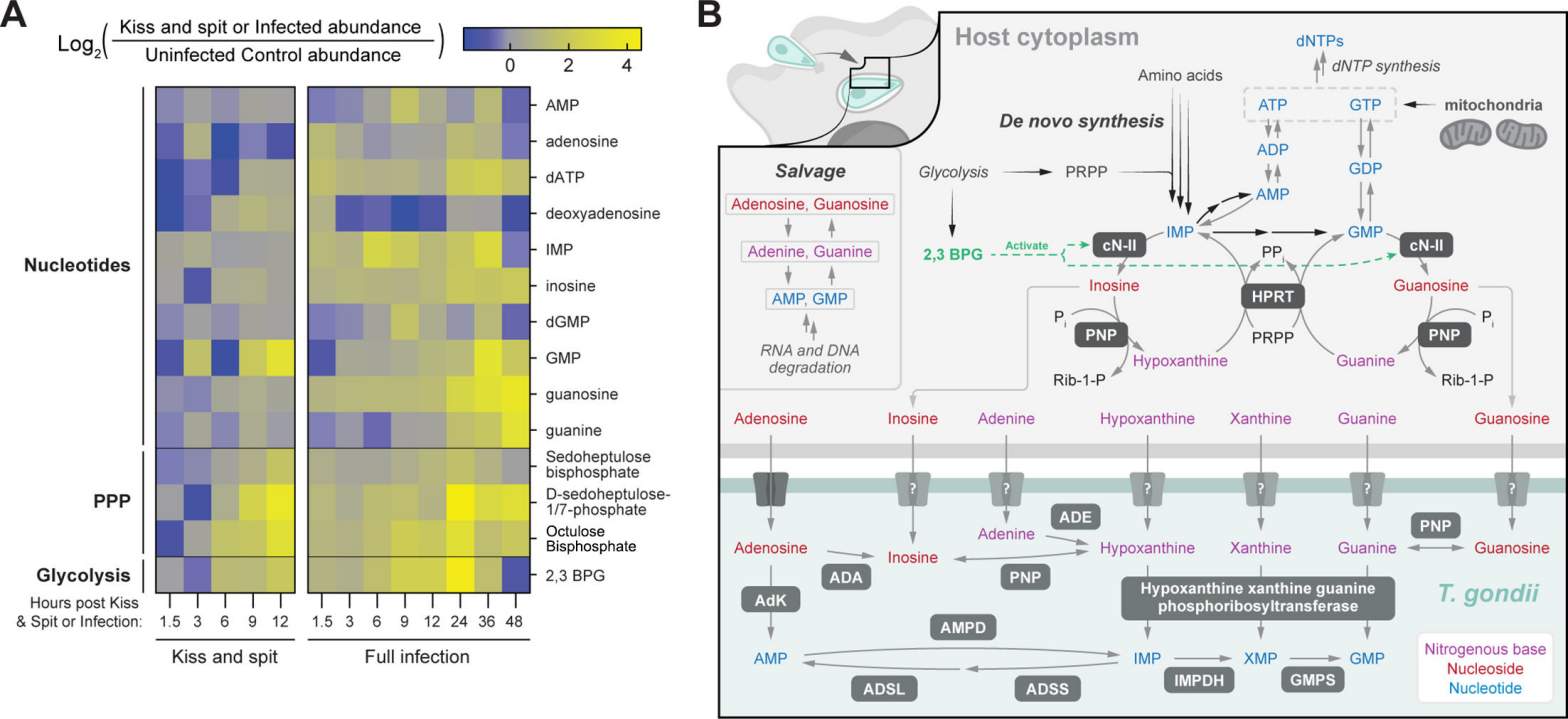
Selected conserved shifts in nucleotide metabolism for full infection and kiss and spit. (**A**) Heatmap of metabolites that are significantly up- (yellow) or downregulated (blue) in response to kiss and spit (left panel) or full infection (right panel). Relative abundances were calculated using kiss and spit or full infection sample abundances, averaged and normalized to the average uninfected control abundance then log base two transformed [log_2_ (kiss and spit or full infection abundance / average control abundance)]. The hours post-infection are shown across the bottom of the heatmap, 1.5, 3, 6, 9, 12, 24, 36, or 48; *N* = 3 for each time point. Metabolites were classified in nucleotides, PPP, and glycolysis. Values represented in this heatmap and their *P*-value (calculated by multiple *t*-test) can be found in Table S2C; the complete metabolomics analysis can be found in Fig. S4. (**B**) The hypothesis of how *T. gondii* infection stimulates the activity of host cytosolic nucleotidase II enzyme (cN-II). *T. gondii* kiss and spit and full infection produce abundant host 2,3-BPG. 2,3-BPG acts as an activator of the host cN-II enzyme. cN-II hydrolyzes nucleotide monophosphates such as GMP and IMP to produce guanosine and inosine, respectively. These nucleosides are then transported to the interior of the parasite cytosol to produce RNA molecules. Adenosine kinase (AdK) and hypoxanthine xanthine guanine phosphoribosyltransferase (HXGPRT) represent the major pathways for salvage and incorporation into the *Toxoplasma* nucleotide pool. Figures modified from Pesi et al. (28, 81). cN-II, cytosolic 5′-nucleotidase II; PNP, purine nucleoside phosphorylase; HPRT, hypoxanthine guanine phosphoribosyl transferase; XO, xanthine oxidase; ADA, adenosine deaminase; ADE, adenine deaminase; ADSL, adenylosuccinate lyase; ADSS, adenylosuccinate synthetase; AMPD, AMP deaminase; GMPS, GMP synthetase; HXGPRT, hypoxanthine_xanthine_guanine phosphoribosyltransferase; IMPDH, inosine 50-monophosphate dehydrogenase; PNP, purine nucleoside phosphorylase; PRPP, 5-phosphoribosyl-1-pyrophosphate.

To understand the differences between full infection and kiss and spit, we compared our previously published metabolomic analysis of full infection (39) and our current kiss and spit metabolomic data set (Fig. 3A; Fig. S4). Over 70 metabolites were identified in both processes (Fig. S4; Table S2), and we selected 14 metabolites that have changes in kiss and spit and full infection: AMP, adenosine, dATP, deoxyadenosine, IMP, inosine, dGMP, guanosine, guanine, SBP, S7P, OBP, and 2,3-BPG. SBP and S7P were significantly more abundant during kiss and spit than in full infection at 12 HPK&S. While S7P is an intermediate in the PPP that generates pentoses and ribose 5-phosphate for nucleotide synthesis, SBP cannot be easily used by the host because it does not contain the required bisphosphatase to dephosphorylate SBP (SBPase) (39). SBPase is a plant-derived gene involved in photosynthesis, absent in human cells but present in *T. gondii*. SBPase greatly increases *T. gondii* ribose metabolism flexibility because it gives the parasite a second energetically driven, but NADP^+^-independent, ribose synthesis pathway (39).

### Kiss and spit increases 2,3-BPG abundance and is related to purine metabolism

2,3-BPG abundance was significantly increased at 9 and 12 HPK&S (Fig. 1B, 2, and 3A; Table S2). 2,3-BPG is not part of the normal glycolytic pathway and is a known allosteric regulator. 2,3-BPG is synthesized in the Rapoport-Luebering shunt, a two-step pathway around the phosphoglycerate kinase step in glycolysis (37). It has already been shown that 2,3-BPG is an allosteric activator of cN-II (38, 82). We hypothesized that high levels of 2,3-BPG upregulate cN-II activity, which in turn generates purine nucleosides for uptake by *T. gondii* (38, 82) (Fig. 3B).

cN-II catalyzes both the hydrolysis of several nucleoside monophosphates and the phosphate transfer from a nucleoside monophosphate donor to the 5′ position of a nucleoside acceptor (26, 27, 29) (Fig. 3B). cN-II acts on substrates such as IMP, GMP, and their corresponding deoxy-derivatives, producing inosine and guanosine, respectively (26, 27, 29). Inosine and guanosine abundance increased not only during *T. gondii* infection but also after kiss and spit (Fig. 2 and 3B). The gene expression analysis of host cN-II and bisphosphoglycerate mutase (BPGM), the enzyme responsible for most of the 2,3-BPG synthesis*,* shows that both enzymes are approximately two fold more expressed during early *T. gondii* full infection (39).

### Host cN-II activity increases in the early hours post *T. gondii* infection and kiss and spit

To evaluate the enzymatic activity of cN-II within *T. gondii*-infected cells and kiss and spit, we used the traditional model of nucleotidase activity assay (83–86). Nucleotidase activity was evaluated by measuring the inorganic phosphate release upon hydrolysis of IMP or GMP (the substrates of the reaction) by cN-II enzyme in protein lysates derived from *T. gondii*-infected, kiss and spit, and uninfected cells. IMP and GMP were used as substrates for the enzymatic reaction at 50 µM.

**Fig 4:**
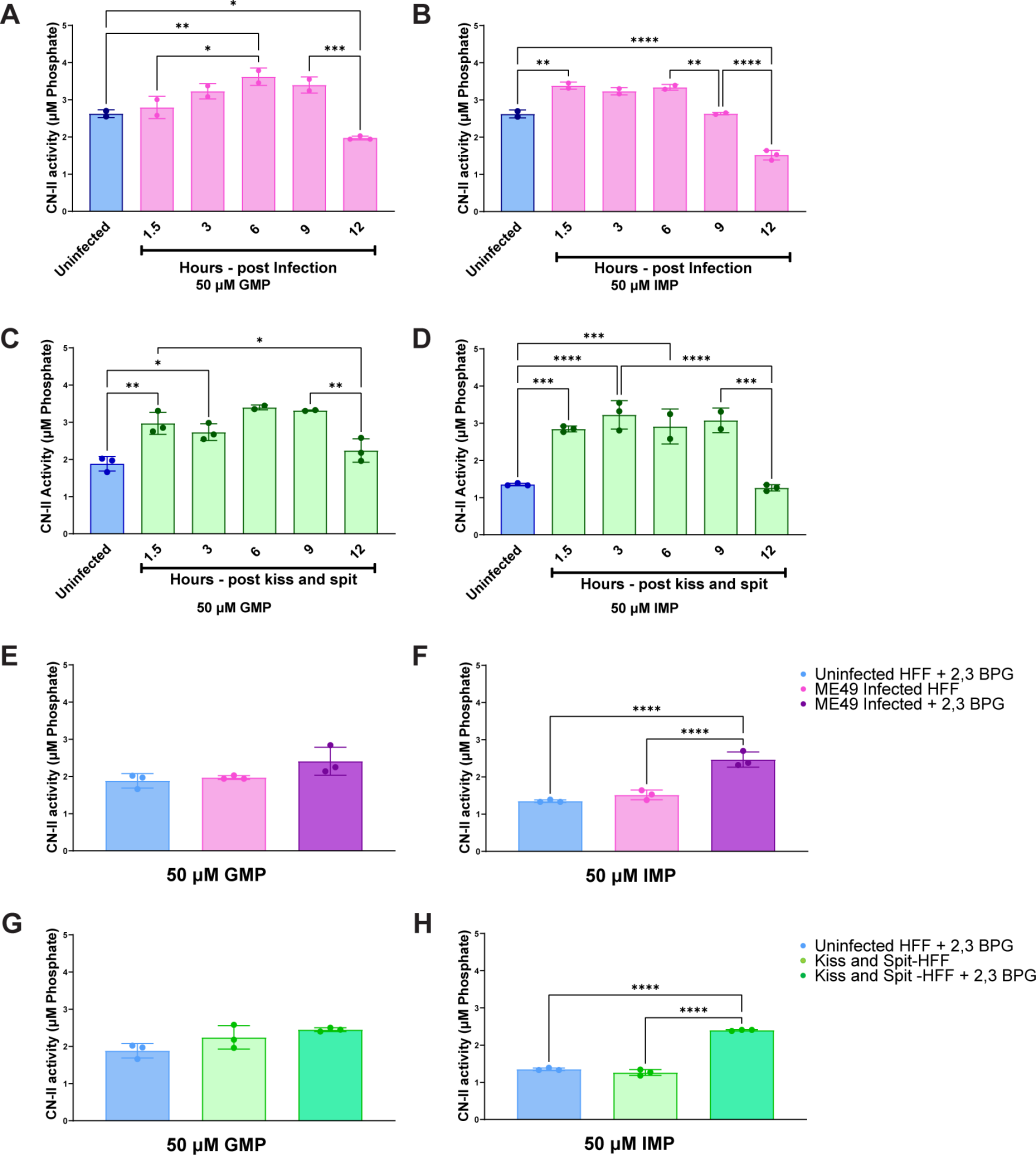
cN-II nucleotidase activity in *T. gondii* fully infected and kiss and spit HFF cells: the cN-II activity was measured in protein lysates according to inorganic phosphate release (µM) using a Malachite Green assay in two independent experiments. IMP and GMP were used as substrates for the enzymatic reaction at 50 µM. (**A**) Uninfected HFF cells and ME49 *T. gondii*-infected HFF cells at different HPI and GMP as substrate. (**B**) Uninfected HFF cells and ME49 *T. gondii*-infected HFF cells at different HPI and IMP as substrate. (**C**) Uninfected HFF cells and ME49 *T. gondii* kiss and spit HFF cells at different hours post kiss and spit and GMP as substrate. (**D**) Uninfected HFF cells and ME49 *T. gondii* kiss and spit HFF cells at different hours post kiss and spit and IMP as substrate. (**E**) Uninfected HFF cells, ME49 *T. gondii*-infected HFF cells at 9 HPI, GMP as substrate, and addition of 2,3-BPG. (**F**) Uninfected HFF cells, ME49 *T. gondii*-infected HFF cells at 9 HPI, IMP as substrate and addition of 2,3-BPG. (**G**) Uninfected HFF cells, ME49 *T. gondii* kiss and spit HFF cells at 9 HPI, GMP as substrate, and addition of 2,3-BPG. (**H**) Uninfected HFF cells, ME49 *T. gondii* kiss and spit HFF cells at 9 HPI, IMP as substrate and addition of 2,3-BPG. Each graph bar represents the mean of *n* = 3 replicates, and error bars represent the SD. Statistical analysis was performed by one-way ANOVA with Tukey’s test to compare conditions. Alpha = 0.05; 0.1234 (ns), 0.0332 (*), 0.0021 (**), 0.0002 (***), <0.0001 (****).

First, we evaluated cN-II activity in HFF cells subjected to full infection with the ME49 strain of *T. gondii* at different time points with GMP (Fig. 4A) or IMP as substrate (Fig. 4B). cN-II activity significantly increased in *T. gondii*-infected cells from 1.5 to 9 HPI but was low at 12 HPI, which correlates with the cN-II expression level reported previously by RNA sequencing (39). Second, we evaluated cN-II activity in HFF cells subjected to kiss and spit with the ME49 strain of *T. gondii* at different time points spit with GMP (Fig. 4C) or IMP as substrate (Fig. 4D). cN-II activity significantly increased in *T. gondii* kiss and spit cells from 1.5 to 9 HPK&S, but cN-II activity was low at 12 HPK&S, which correlates with the guanosine and inosine levels, products of these enzymatic reactions (Fig. 1 and 3A). Then, we evaluated uninfected HFF cells, ME49 *T. gondii*-infected HFF cells at 9 HPI, GMP as substrate and addition of 2,3-BPG (Fig. 4E) or IMP as substrate and addition of 2,3-BPG (Fig. 4F). Next, we performed the assay on uninfected HFF cells, ME49 *T. gondii* kiss and spit HFF cells at 9 HPI, GMP as substrate and addition of 2,3-BPG (Fig. 4G) or IMP as substrate and addition of 2,3-BPG (Fig. 4H). For both full infection and kiss and spit, the addition of 2,3-BPG increase the cN-II enzymatic activity, confirming its role as allosteric regulator.

### Chemical inhibition of cN-II enzyme reduces *T. gondii* replication

**Fig 5:**
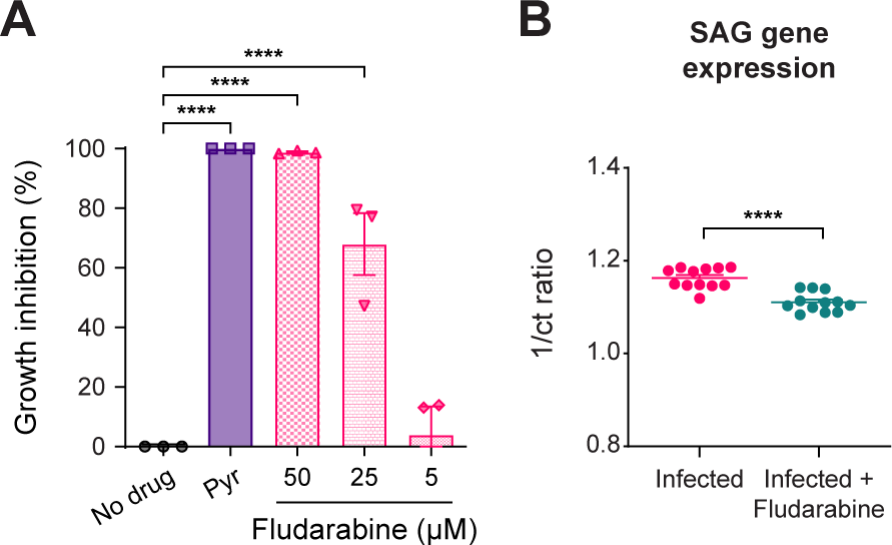
Effect of fludarabine on *T. gondii* replication and cN-II activity: (**A**) fludarabine inhibits *T. gondii* replication rate of [^3^H] uracil uptake under five conditions: no drug treatment negative control for growth inhibition, 1 μM pyrimethamine positive control for total growth inhibition, and either 50, 25, or 5 μM fludarabine treatment. Uninfected cells were also assayed for a baseline level of [^3^H] uracil uptake by host cells. Uracil uptake was measured, host incorporation was subtracted, and each condition was normalized to the uninhibited DMSO control condition to determine percent growth inhibition. Error bars represent the SEM of three replicates per condition. Statistical analysis was performed by one-way ANOVA with Dunnett’s multiple comparisons. Alpha = 0.05. Pairwise comparisons were performed with the negative control no drug. *P* value = 0.0024 for no drug vs pyrimethamine and *P* value = 0.0273 for no drug vs 50 µM. *N* = 3. (**B**) *T. gondii* surface antigen 1 (SAG) gene expression. Gene expressions were evaluated in PruΔHXGPRT-infected HFF cells with and without 50 µM fludarabine at 24 HPI, using human peptidylprolyl isomerase A (PPIA) as the housekeeping gene. Primer sequences are listed in Table S2J. Statistical analysis was performed by *t*-test. Alpha = 0.05; 0.1234 (ns), 0.0332 (*), 0.0021 (**), 0.0002 (***), <0.0001 (****). PruΔHXGPRT-infected HFF cells are in magenta, and PruΔHXGPRT-infected HFF cells + fludarabine are in cyan. *N* = 12.

To assess the effect of host cN-II inhibition on *T. gondii* growth, we performed a [^3^H] uracil uptake assay in the presence of 50, 25, or 5 μM of the cN-II inhibitor fludarabine (Fig. 5A). As a negative control for growth inhibition, *T. gondii* was grown untreated, and as a positive control, parasites were treated with 1 μM pyrimethamine, which is a common treatment for toxoplasmosis. Uninfected host cells were also assayed to determine background host uptake of [^3^H] uracil. We found that fludarabine inhibits *T. gondii* growth in a dose-dependent manner, with 50 μM completely halting replication (Fig. 5A). We also performed quantitative PCR (qPCR) with a parasite gene, surface antigen 1 (SAG), to evaluate replication of the intracellular parasite with and without 50 µM of fludarabine. The parasite replication was significantly lower in infected cells treated with fludarabine (Fig. 5B).

### Metabolomics and gene expression analysis of fludarabine-treated *T. gondii*-infected cells show differences in purine metabolism

**Fig 6:**
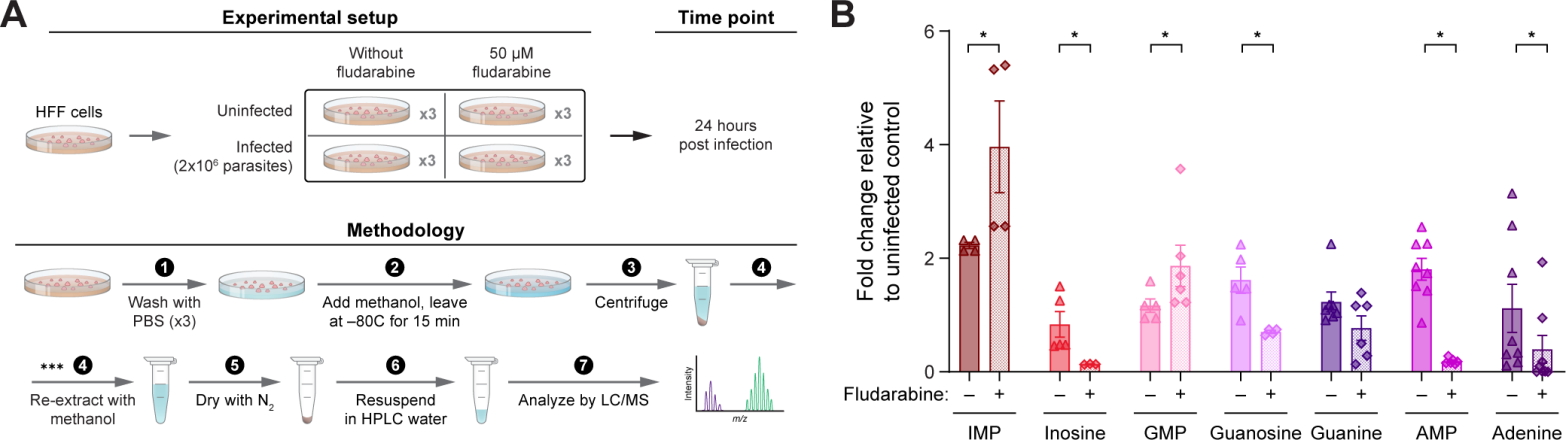
Effect of fludarabine on purine metabolism in *T. gondii*-infected HFF host cells. (**A**) Metabolomic methodology created in BioRender. Lopez, G. (https://BioRender.com/e9zg8be) is licensed under CC BY 4.0. (**B**) Relative abundance of purine metabolites in HFF cell line infected with PruΔHXGPRT *T. gondii* and treated with fludarabine (+) at 24 HPI or without fludarabine (−). Metabolites were quantified using HPLC-MS and identified with known standards. Each bar represents the fold change of the abundance mean of two independent experiments (*N* = 4–9), normalized to the abundance mean of the uninfected control. Statistical analyses were performed by multiple *t*-tests. Alpha = 0.05; 0.1234 (ns), 0.0332 (*), 0.0021 (**), 0.0002 (***), <0.0001 (****). Pru ΔHXGPRT-infected HFF cells are represented by a triangle symbol, and Pru ΔHXGPRT-infected HFF cells + fludarabine are represented by a rhombus symbol. comparison with Pru WT-infected cells in Fig. S5A.

To understand the mechanism of fludarabine growth inhibition, we performed metabolomics of infected HFF cells, comparing infections with the parental Pru WT and PruΔHXGPRT strains in the presence and absence of fludarabine (Fig. 6A). To reduce the effect of purine interconversion in the parasite, we used Pru parasites with a genetic deletion of the hypoxanthine-guanine phosphoribosyl transferase (HXGPRT) enzyme (PruΔHXGPRT). HXGPRT phosphorylates imported purines, but it is not essential for *T. gondii* due to the activity of adenosine kinase (AK) and the conversion of AMP to IMP, XMP, or GMP (87).

Fludarabine affected the abundance of purines in *T. gondii*-infected HFF cells at 24 HPI (Fig. 6B; Fig. S5). IMP and GMP, the preferred substrates of the cN-II enzyme, accumulated with statistical significance when treated with fludarabine in PruΔHXGPRT-infected cells, as we expected (Fig. 6B). The nucleobase products of the cN-II reaction, inosine and guanosine, were significantly less abundant with fludarabine treatment in PruΔHXGPRT-infected cells (Fig. 6B). In Pru WT-infected cells, the effect is not clear, possibly due to the interconversion of purines (Fig. S5A).

**Fig 7:**
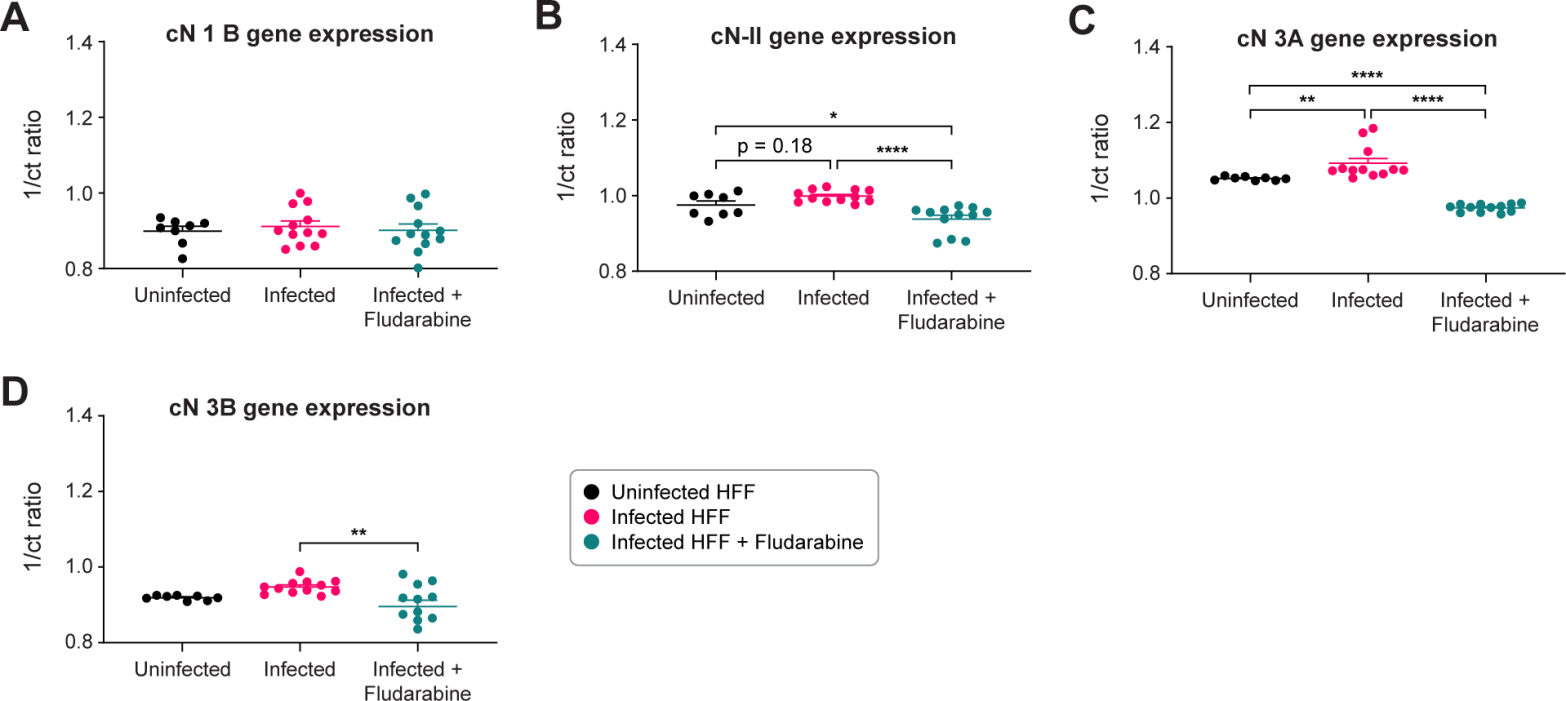
Host cytosolic nucleotidase enzymes gene expression during *T. gondii* infection and fludarabine treatment. (**A**) Host cytosolic nucleotidase 1B (cN-1B) gene expression. (**B**) Host cN-II gene expression. (**C**) Host cN-3A gene expression. (**D**) Host cN-3B gene expression. Gene expressions were evaluated in three conditions: uninfected HFF cells and PruΔHXGPRT-infected HFF cells with and without fludarabine at 24 HPI, using human PPIA as the housekeeping gene. Primer sequences are listed in Table S2. Statistical analysis was performed by one-way ANOVA, and multiple comparison performed by Tukey’s test. Alpha = 0.05; 0.1234 (ns), 0.0332 (*), 0.0021 (**), 0.0002 (***), <0.0001 (****). Uninfected cells are represented in black color, PruΔHXGPRT-infected HFF cells are in magenta, and PruΔHXGPRT-infected HFF cells + fludarabine are in cyan. Primer sequences are in Table S2J. *N* = 8–12.

We performed qPCR to measure expression levels of different enzymes in uninfected and infected HFF cells with and without fludarabine. cN-1B expression did not show any significant changes with infection or fludarabine treatment (Fig. 7A). CN-II enzyme tended to be upregulated with infection, but it was not significant by qPCR (*P* = 0.18) (Fig. 7B). However, this upregulation was previously observed by RNA sequencing analysis (39). We found that *T. gondii* infection significantly increased the expression of host cN-3A (Fig. 7C). We found that in PruΔHXGPRT-infected HFF cells treated with fludarabine, there was a significant decrease in the expression of host cN-II, cN-3A, and cN-3B enzymes (Fig. 7B through D).

It is possible that AMP, the preferred substrate for cN-I and the adenine phosphoribosyltransferase (APRT) enzyme, is lower in PruΔHXGPRT-infected HFF cells treated with fludarabine (Fig. 6B) because the parasites are using more adenine to compensate for inosine and guanosine reduction. However, according to our qPCR for cN-1B (Fig. 7A), the expression level of host cN-1 did not change significantly during infections or the treatment with fludarabine. This finding suggests adenine may be produced more abundantly from AMP via the APRT enzyme (88, 89) to compensate for inosine and guanosine, but we did not detect adenosine in all samples. Thus, it is likely that fludarabine inhibits the cN-II enzyme, which affects the metabolites IMP, GMP, inosine, and guanosine, and reduces the replication of the parasite in infected cells.

It is likely that nucleoside analogs, structurally similar to the substrate of cytosolic nucleotidase enzymes, could act as inhibitors of these enzymes by binding to their active or regulatory site (90). It is possible that the accumulation of nucleotide monophosphates could result in feedback inhibition of host enzymes such as cN-3A and cN-3B in the PruΔHXGPRT parasite strain, which depends more heavily on inosine/adenine salvage.(Fig. 7C and D). Many potential inhibitors of cytosolic nucleotidase enzymes have been designed as nucleoside analogs of the substrates and used as drug treatments for different diseases (84, 86, 90–92).

### Deletion of cN-II affects replication of parasite and purine metabolism

**Fig 8:**
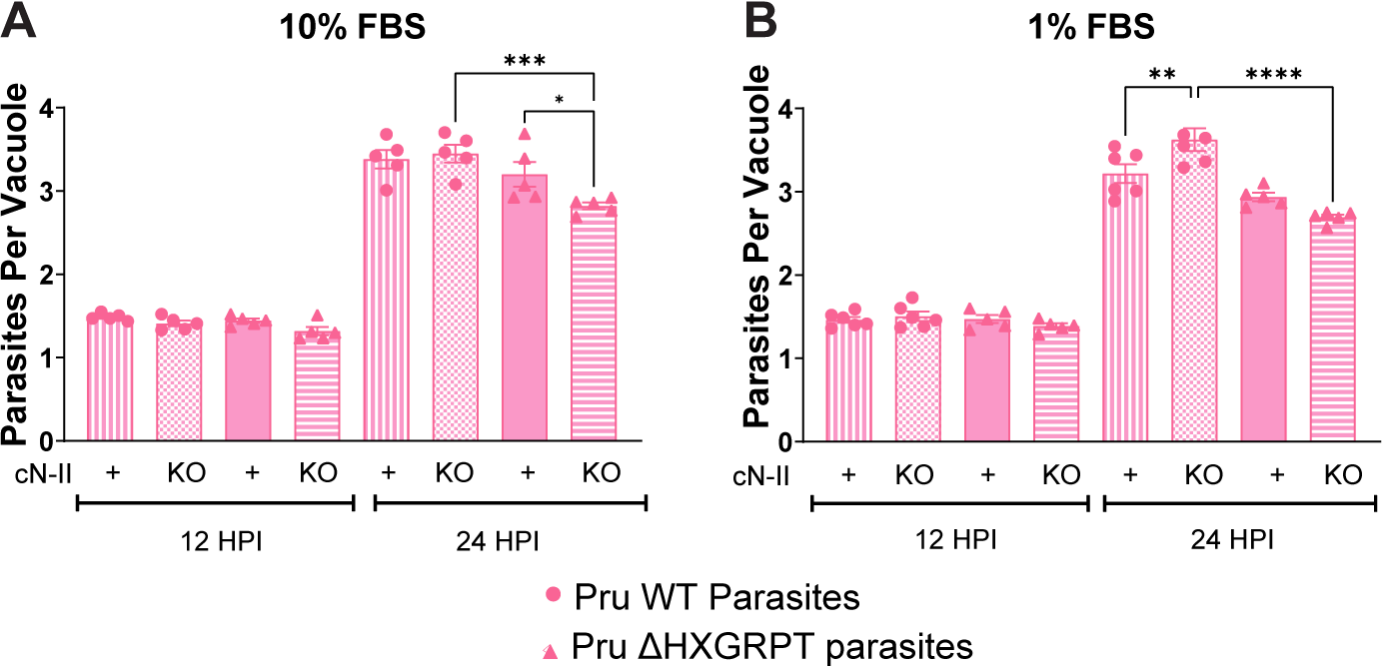
Effect of cN-II gene deletion in *T. gondii*-infected cells. (**A**) Intracellular parasites grew in media with 10% FBS, and (**B**) intracellular parasites grew in media with 1% FBS. Detailed parasite per vacuole counts can be found in Fig. S5B through E. Statistical analyses were performed by one-way ANOVA; 0.1234 (ns), 0.0332 (*), 0.0021 (**), 0.0002 (***), <0.0001 (****). Infected MDAMB231 cells are represented with (+) and infected MDAMB231 cN-II KO cells are represented with KO. Pru WT parasites are represented by circular symbols, and Pru-ΔHXGPRT represented by triangle symbols. Each dot represents an independent replicate, the graph bar shows the average of six replicates, and the error bars represent the SEM.

To confirm the role of the cN-II enzyme in purine metabolism in *T. gondii* infection, we took advantage of the existence of a cN-II knockout in a breast cancer cell line denominated MDAMB231 (85, 93). First, we evaluated the effect of host cN-II genetic deletion on the replication of the intracellular parasite. The parasite per vacuole counts in MDAMB231 parental cell line vs MDAMB231 cN-II KO using Pru WT and PruΔHXGPRT parasites showed that the deletion of the host cN-II enzyme slightly reduced the replication of the parasite at 12 HPI and significantly reduced replication at 24 HPI in 10% fetal bovine serum (FBS) media and 1% FBS media (Fig. 8; Fig. S5B through E).

**Fig 9:**
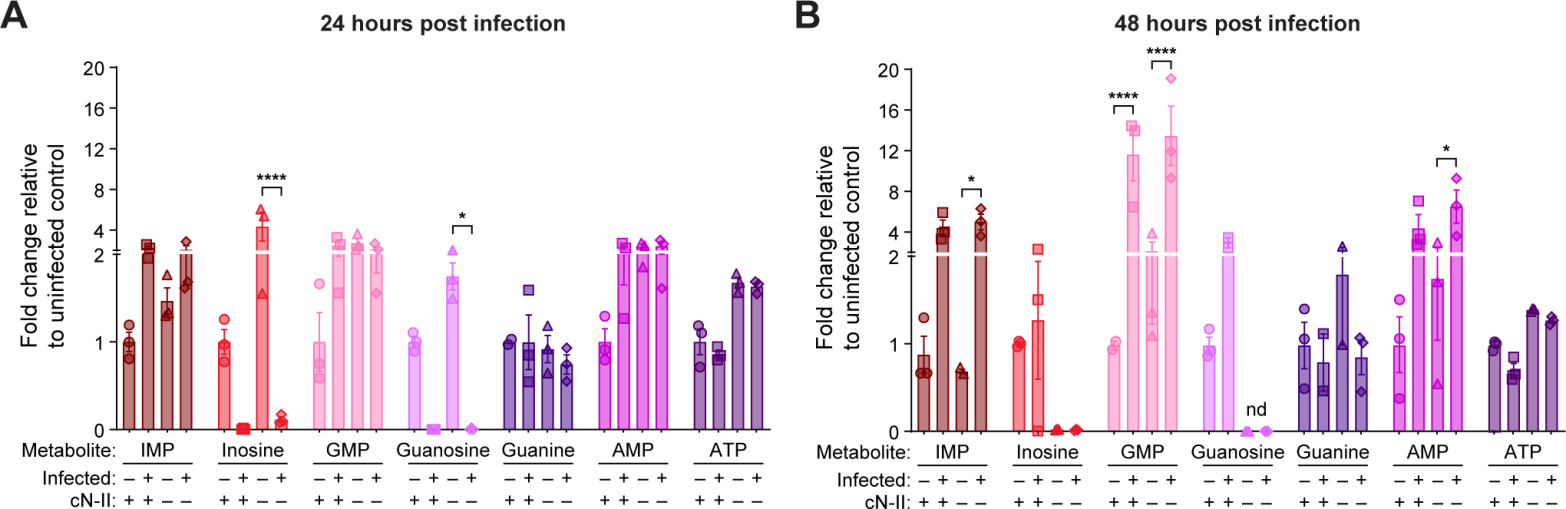
Effect of cN-II gene deletion on purine metabolism in *T. gondii*-infected host cells. Relative abundance of purine metabolites in MDAMB231 cells (+) and in MDAMB231 cN-II knockout cells (−), uninfected (−) and infected with ME49 *T. gondii* (+), (**A**) at 24 HPI, (**B**) at 48 HPI. Metabolites were quantified using HPLC-MS and identified with known standards. Each graph bar represents the mean of the fold change of selected metabolites in infected cells with respect to their respective uninfected MDAMB231 control. Each graph bar represents the mean of three replicates, and error bars represent the SEM. Statistical analysis was performed by two-way ANOVA with Bonferroni’s test to compare conditions in each metabolite. Alpha = 0.05; 0.1234 (ns), 0.0332 (*), 0.0021 (**), 0.0002 (***), <0.0001 (****). Uninfected MDAMB231 cells are represented with − +; ME49-infected MDAMB231 cells are represented with ++; uninfected MDAMB231 cN-II knockout cells are represented with − −; and ME49-infected MDAMB231 cN-II knockout cells are represented with + −. Nd, not detected.

To understand the effect of genetic deletion of host cN-II in *T. gondii* infection, we performed metabolomics on the uninfected and *T. gondii-*infected MDAMB231 parental and cN-II KO cell lines. IMP and GMP, the preferred substrates of the cN-II enzyme, accumulated in infected cells in comparison to uninfected cells, but the effect was significant in MDAMB231 cN-II KO cells at 48 HPI (Fig. 9). The nucleobase products of the cN-II reaction, inosine and guanosine, were significantly less abundant at 24 HPI in infected cells (Fig. 9A); at 48 HPI, inosine was very low in abundance and guanosine was not detected in cN-II KO cell lines (Fig. 9B). AMP was significantly more abundant with infection in MDAMB231 cN-II KO cells. Thus, the genetic deletion of the cN-II enzyme affected the metabolites IMP, GMP, inosine, guanosine, and AMP in infected cells.

### AMP compensates for the purine metabolism in ME49 *T. gondii*-infected host cells

**Fig 10:**
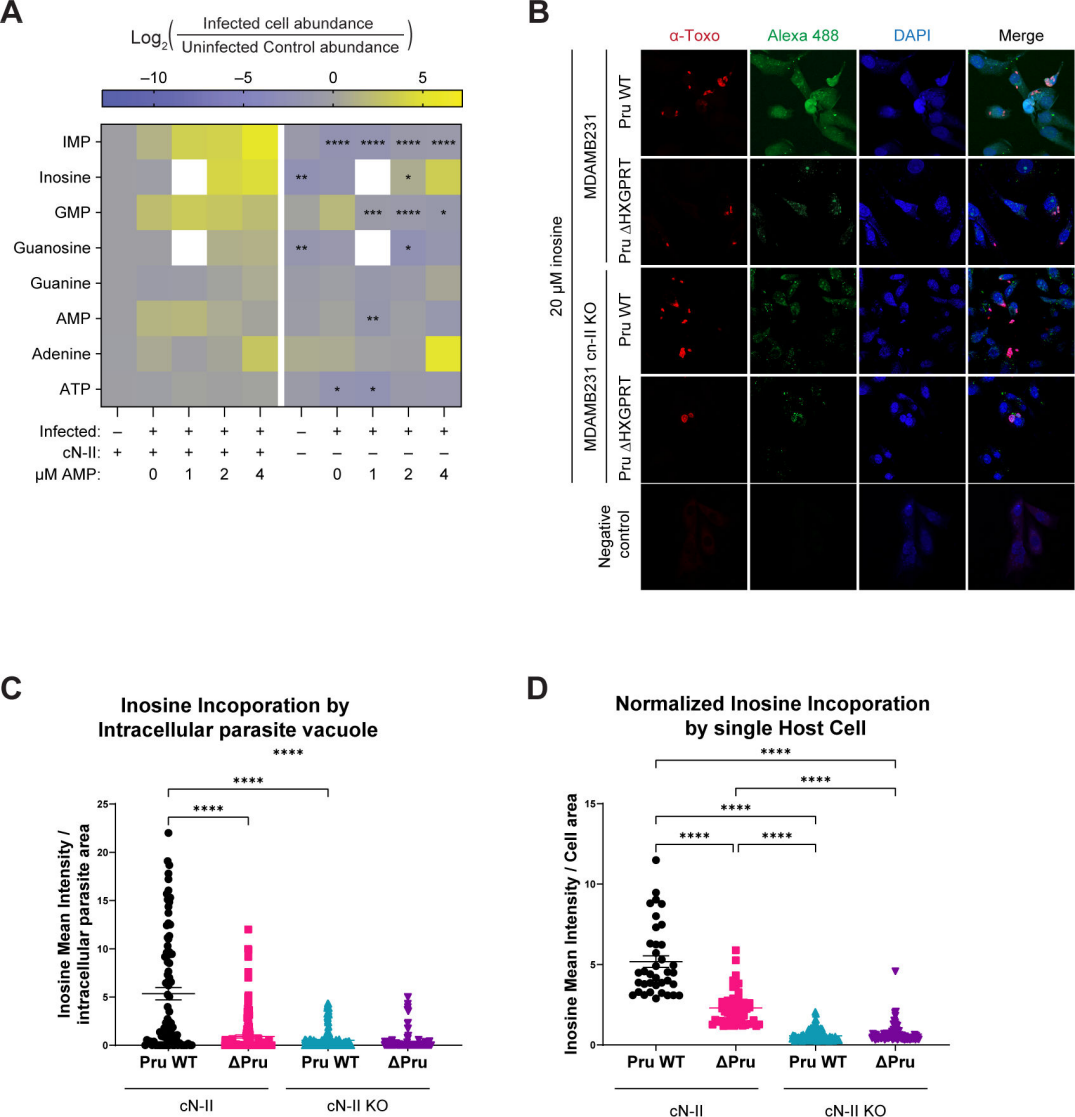
Effect of AMP and inosine addition on purine metabolism in *T. gondii*-infected host cells. (**A**) Heatmap of selected purine metabolites in MDAMB231 cell line (+), in MDAMB231 cN-II knockout cells (−), uninfected (−), and infected with ME49 *T. gondii* (+), and in increasing addition of AMP (0, 1, 2, 4 µM) at 48 HPI. Scales show the log_2_ fold change in infected cells with respect to the uninfected control, with blue being less abundant and yellow more abundant. Metabolites were selected for inclusion based on whether they could be confidently identified with the standards. *N* = 3. Statistical analysis was performed by two-way ANOVA with Bonferroni’s multiple comparisons test to compare MDAMB231-cN-II knockout cells vs MDAMB231 in each condition and in each metabolite. Alpha = 0.05; 0.1234 (ns), 0.0332 (*), 0.0021 (**), 0.0002 (***), <0.0001 (****). Values represented in this heatmap, and *P*-values can be found in Table S2D; a more detailed graph can be found in Fig. S6. (**B**) Clickable purine analysis to visualize inosine incorporation on infected MDAMB231 cells with and without genetic deletion of cN-II enzyme. Inosine (EdI) incorporation and Cu-catalyzed azide-alkyne staining of active intracellular parasites in MDAMB231 parental, and MDAMB231 cN-II KO host cells infected with Pru-WT or PruΔHXGPRT parasites at 48 HPI. EdI was added to 20 µM in cell culture media. α-*Toxoplasma* localizes in red the *T. gondii* parasite detected with chronic *T. gondii*-infected mice serum. Alexa 488 localizes in green the incorporation of EdI. 4′,6-Diamidino-2-phenylindole (DAPI) staining localizes in blue the cell and parasite nuclei. Merged channels localize the morphology of both cells and parasites. The representative images displayed in this figure were increased in resolution for better visualization. (**C**) Quantification of inosine incorporation by intracellular parasite vacuole. *N* = 89–180. (**D**) Quantification of inosine incorporation by single host cell normalized to the negative control. *N* = 30–866. All images quantified were acquired using the same setting in the microscope without any alteration. Intensity = photon number detected in each pixel. Area = pixel number in each single cell. Each dot represents a single parasite vacuole (left) or a single cell (right), and error bars represent the SEM. Statistical analysis was performed by one-way ANOVA with Tukey’s correction for multiple comparisons. Alpha = 0.05; 0.1234 (ns), 0.0332 (*), 0.0021 (**), 0.0002 (***), <0.0001 (****). Pru-WT infected MDAMB231 cells are represented in black, Pru ΔHXGPRT infected MDAMB231 cells are in fuchsia, Pru-WT infected MDAMB231 cN-II KO cells are in cyan, and PruΔHXGPRT infected MDAMB231 cN-II KO cells are in purple.

*T. gondii* preferentially uses the AMP salvage pathway to obtain purines (81). Parasites treated with fludarabine or with genetic deletion of cN-II may use AMP to compensate for the inosine and guanosine reduction. AMP is the preferred substrate for cN-I and the APRT enzyme. However, according to our qPCR for cN-1B (Fig. 7A), the expression level of host cN-1B did not change significantly with infection or with fludarabine treatment. An alternative hypothesis is that adenine is produced more abundantly from AMP via the APRT enzyme to compensate for inosine and guanosine reduction (88, 89). We performed targeted metabolomics in uninfected and infected MDAMB231 parental and MDAMB231 cN-II KO cells to study purine metabolism compensation at different concentrations of AMP (Fig. 10A; Fig. S6).

The results suggest that AMP contributed to the abundance of IMP, inosine, guanosine, guanine, ATP, and adenine in both infected MDAMB231 parental and cN-II KO cell lines, though with varying statistical significance. We found significant differences in MDAMB231 cN-II KO cell lines with respect to the MDAMB231 parental cell line with supplementation of AMP in the metabolites IMP, inosine, GMP, guanosine, AMP, and ATP (Fig. 10A). With increasing concentrations of AMP, the metabolites IMP, guanosine, and AMP significantly accumulated more in infected MDAMB231 parental cells. This accumulation was also seen in infected MDAMB231 cN-II KO cells but did not meet the threshold of statistical significance (Fig. S6A, D, and F). Adenine significantly accumulated more in infected MDAMB231 cN-II KO cells with increasing concentration of AMP, and again in infected MDAMB231 parental cells but without statistical significance (Fig. S6B and H). This finding suggests a compensatory effect of AMP and adenine in purine metabolism in *T. gondii*-infected cells is greater when host cN-II enzyme is deleted.

Adenosine was not detected in the metabolomics data, perhaps because the pool of adenosine in host cells is very low (~1 µM) (94–96). Guanine accumulated with increasing concentration of AMP in both infected MDAMB231 parental cells and in infected MDAMB231 cN-II KO cells (Fig. S6E), though not significantly. Finally, GMP and AMP both significantly accumulated with 1 µM of AMP in infected MDAMB231 parental cells but reduced in abundance with 2 and 4 µM of AMP. In infected MDAMB231 cN-II KO cells, both GMP and AMP significantly reduced in abundance with compensation of AMP (Fig. 10A; Fig. S6C and G). This lower abundance of AMP with 2 or 4 µM AMP supplementation probably is due to the interconversion of the purines from AMP toward IMP, inosine, guanosine, guanine, and adenine.

### Click chemistry tracks *T. gondii* purine acquisition

We used click chemistry to evaluate our hypothesis that *T. gondii* uses the host cN-II enzyme to produce purines from GMP or IMP. MDAMB231 parental cells and MDAMB231 cN-II KO cells were cultured in normal media and then changed to media depleted of purines just before the experiment. We added inosine containing an inosine-alkyne modified terminal group (EdI). At 48 HPI, the cells were fixed, permeabilized, and incubated with the clickable reagent, which has the azide component for the click chemistry. We observed EdI incorporation in both intracellular parasites and host cells (Fig. 10B; Fig. S7). The least EdI incorporation in both host and parasite was observed in PruΔHXGPRT-infected MDAMB231 cN-II KO host cells (Fig. 10B through D; Fig. S7). More parasite EdI incorporation was observed when MDAMB231 parental host cells were infected with Pru WT or PruΔHXGPRT parasites. The maximum EdI incorporation was observed when MDAMB231 parental host cells were infected with Pru WT parasites (Fig. 10D; Fig. S7). These results suggest that the genetic ablation of cN-II enzyme in host cells and the genetic ablation of HXGPRT in parasites result in less incorporation of EdI. If cN-II dephosphorylates IMP and GMP to produce inosine and guanosine, then in the absence of cN-II enzyme, the host cell and intracellular parasite incorporated less EdI from the media because it does not have the host enzymatic machinery to process it. Thus, PruΔHXGPRT parasites induce or require less incorporation of the dephosphorylated EdI from the media.

The host cell phosphorylation/dephosphorylation cycles catalyzed by cN-II affect the cell nucleoside pool, as has been reported previously (24, 97). Thus, cN-II regulates inosine homeostasis, as has been demonstrated in *Saccharomyces cerevisiae* (98). Additionally, it has been demonstrated that nucleoside analogs are phosphorylated intracellularly by the same kinases involved in substrate cycles, and the resulting monophosphates may be dephosphorylated by intracellular 5′-nucleotidase activities (24, 99). Thus, the mechanism we proposed is that when cN-II is present, EdI is accumulated. However, when cN-II is knocked out, EdI is consumed to produce other purines, making them available for parasite import.

## DISCUSSION

*T. gondii* is an obligatory intracellular parasite that cannot replicate outside of its host cell and can only exist in extracellular forms for a short time without any replication (81). *T. gondii* can produce pyrimidines *de novo* but cannot produce purines (100), and instead relies on its host (101, 102) (Fig. 3B). These nucleotides are essential to cellular functions that involve DNA and RNA synthesis, chemical energy (ATP), nucleotide-based enzyme cofactors (NAD, FAD), and secondary messengers (cAMP) (81, 103). In *T. gondii,* three purine transporters have been characterized so far, TgAT1, TgAT2, and TgNT1 (81) (Fig. 3B). Ultimately, the identification of host enzymes used by the parasite in purine salvage is essential for designing better drugs against parasites.

It is challenging to study purine metabolism in apicomplexan parasites because it is difficult to separate the parasite from the host cell. The capture, transport, and salvage pathways necessary for purine acquisition have long been viewed as an Achilles’ heel of the parasite that may be targeted for chemotherapy (81). Current treatment strategies in human infections caused by *T. gondii* or *P. falciparum* are based on targeting parasite enzymes and blocking the accumulation of nucleotides. This validated approach to chemotherapy highlights the significance of further research to dissect the details of nucleotide metabolism in the Apicomplexa (81). Our work suggests that one of the pathways that *T. gondii* uses to produce nucleoside pools is the hijacking of the host cN-II enzyme. *T. gondii* does not internalize host cN-II as a functional enzyme in its cytoplasm. The parasite is dependent on cN-II for its salvage of precursors for its own pathways. cN-II dephosphorylates IMP and GMP, toward inosine and guanosine respectively, which parasite cannot synthetize and require internalize for its replication because it cannot transport with high efficiency the host phosphorylated precursors.

Kiss and spit (Fig. 1A) is a process where the *T. gondii* parasite secretes rhoptry proteins into the host cell (41, 104). After kiss and spit occurs, the parasite uses actin polymerization to enter the host cell (40). Treating parasites with the actin polymerization inhibitor Cyt D allows kiss and spit to occur without invasion and replication (51, 52). During Cyt D treatment, the secretion of micronemes and rhoptry proteins and formation of parasitophorous vacuoles still occurs (41). However, the release of dense granule markers is blocked by Cyt D (41). For this reason, Cyt D treatment leads to the formation of evacuoles, protein-rich vesicles in the host cell cytosol that lack an internal parasite (105, 106). Cyt D treatment does not affect cell attachment (48) but blocks gliding motility, disrupting parasite actin filaments (40, 47, 107).

We discovered that kiss and spit changes host metabolite abundance in multiple pathways including glycolysis, PPP, amino acid synthesis, gluconeogenesis, and nucleotide synthesis (Fig. 1 and 2A; Fig. S1A and S3A). These metabolomic analyses highlight how microbial secreted contents remodel the host cell metabolism even without invasion. The metabolic regulation by the kiss and spit mechanism is indispensable for the survival of the parasite as purines are essential for the parasites within successfully invaded host cells. We have already demonstrated that kiss and spit affects the redox biology of the host cell (104), and a prior study demonstrated in an *in vivo* mouse model that *T. gondii* manipulates uninfected–injected (or “kiss and spit”) cells much like infected–injected cells (108). Future studies are needed to define the precise *T. gondii* molecules in the rhoptries (109) and the dense granule proteins that are responsible for manipulating host enzymes (46).

Our previous metabolomic profile of full infection (39) and the current kiss and spit metabolomic profile show that the parasite changes the abundance of many host metabolites related to nucleotide synthesis such as SBP, S7P, OBP, 2,3-BPG, and the purines inosine, guanosine, GMP, AMP, and IMP (Fig. 3A). A surprising finding from the kiss and spit metabolome was the quadrupling of host 2,3-BPG abundance at 12 HPK&S (Fig. 1). Host BPGM, the main source of 2,3-BPG, is transcriptionally upregulated in infected host cells during the first nine hours of infection (39). *T. gondii* BPGM has yet to be identified, which makes it difficult to estimate the parasite’s contribution to 2,3-BPG synthesis. In this study, we tested the hypothesis that kiss and spit increases 2,3-BPG synthesis which then acts as an allosteric activator of the cN-II enzyme, which in turn generates purine nucleosides for *T. gondii* (Fig. 3B).

Gene expression analysis of *T. gondii* full infection in HFF cells showed a high abundance of the host’s cN-II (39). cN-II gene expression varies among different cells, but it is highly expressed in thyroid, testis, bladder, and esophagus (110). Previous research has demonstrated that cN-II activity is modulated by substrate binding and by effector molecules such as 2,3-BPG, ATP, and GTP in an allosteric site (29, 111). cN-II dephosphorylates the purines IMP, GMP (and sometimes XMP) to inosine, guanosine, and xanthine, respectively (26, 27, 29). cN-II enzymes are present not only in human cells but are also present in other eukaryote and prokaryote organisms. PfISN1 is the first 5′ nucleotidase reported in *Plasmodium falciparum,* which is an IMP-specific nucleotidase that is allosterically activated by ATP (20). A human ecto-nucleotidase has also been reported to produce adenosine from AMP in the *T. gondii* infection context (90, 112).

Using chemical or genetic inhibition of the cN-II enzyme in *T. gondii*-infected cells, we observed (i) accumulation of cN-II substrates such as the nucleotides GMP and IMP, (ii) reduction of the intracellular abundance of the nucleobase products of this reaction such as guanosine and inosine, respectively, and (iii) reduction of the abundance of the nucleobase subproducts of this reaction such as guanine (Fig. 6B and 9). *T. gondii* uses different routes to obtain purines, including salvaging host materials and interconverting purines inside the parasite (19, 103) (Fig. 3B). *T. gondii* can transport and salvage the host nucleosides adenosine, inosine, and guanosine, as well as the host nucleobases adenine, hypoxanthine, xanthine, and guanine. At least nine enzymes have been reported in *T. gondii* to be involved in both interconversion and salvage of host purines (6) (Fig. 3B), which explains our results in Pru WT strain (Fig. S5A) and AMP compensation (Fig. S6).

Among the enzymes related to purine interconversion, *T. gondii* adenosine kinase (TgAK) is the main route of purine incorporation in the parasite (6). TgAK uses host adenosine to form the nucleoside AMP in the interior of the parasite, and parasites lacking TgAK can survive due to the presence of the HXGPRT enzyme. *T. gondii* salvages hypoxanthine, xanthine, and guanine through HXGPRT by converting them into their respective nucleosides (6). TgHXGPRT has two isoforms encoded by a single gene and with two different intracellular localizations (113). AK and HXGPRT cannot be genetically disrupted simultaneously, suggesting that these routes are essential for the parasite’s purine metabolism and survival (6). *T. gondii* has a redundant and compensatory purine salvage pathway using AK and HXGPRT enzymes. When we used a PruΔHXGPRT parasite, we saw fludarabine inhibition of the host cN-II enzyme, observed by the accumulation of cN-II substrates and reduction of cN-II products (Fig. 6B). Our results using clickable EdI chemistry add evidence to our hypothesis that *T. gondii* uses the host cN-II enzyme to produce purines from GMP or IMP (Fig. 10B).

We used fludarabine for the chemical inhibition of host cN-II; however, it may also be affecting directly the parasite. Fludarabine is a nucleoside prodrug that enters the cell and accumulates mainly as the phosphorylated form, F-ara-ATP, which inhibits cN-II, but it also has off-target effects on other enzymes such as adenosine deaminase (114, 115). For this reason, it is possible that fludarabine is affecting the replication of the parasite directly. We did find lower levels of inosine and guanosine, and higher levels of IMP and GMP after fludarabine treatment (Fig. 6B). We also confirmed that fludarabine is functioning as a cN-II inhibitor by qPCR (Fig. 7B). Regardless of whether fludarabine’s anti-*T*. *gondii* mechanism of action is inhibiting the host cN-II enzyme alone, or an additional parasite enzyme(s), this already FDA-approved drug could be considered for combination therapy to treat toxoplasmosis.

## MATERIALS AND METHODS

### *T. gondii* strains and cell culture

Low-passage type II ME49 *T. gondii* was used in all kiss and spit experiments. Pru and PruΔHXGPRT *T. gondii*, a gift from D. Soldati (116), were used for fludarabine metabolomics. HFFs, MDAMB231 parental, or cN-II-KO MDAMB231 cells (donation from Lars Petter Jordheim lab) were grown in DMEM with 10% FBS, 2 mM L-glutamine, and 1% penicillin-streptomycin (Sigma-Aldrich). Once HFFs or MDAMB231 cells were in deep quiescence, defined as 10 days post-confluency, DMEM media was changed to metabolomic media, RPMI 1640 supplemented with 2 mM L-glutamine, 1% FBS dialyzed against PBS (MW cutoff of 10 kDa), 10 mM HEPES, and 1% penicillin-streptomycin. After 35 hours, the media was again changed to metabolic media, 1 hour before treatment with *T. gondii*.

### Kiss and spit time course metabolomics

HFFs were seeded in 60 mm dishes and allowed to reach confluency. Then, HFF dishes in triplicate were treated with 2 × 10^6^ ME49 tachyzoites that had been pre-incubated with Cyt D at a final concentration of 1.5 μM for 15 minutes (Sigma-Aldrich) in 4 mL media per dish. An additional negative control of media only treated with cytochalasin D was added to a separate set of dishes. Cyt D is a reversible actin inhibitor; for this reason, it was kept in the media all the time during the experiments. At time points 1.5, 3, 6, 9, and 12 HPK&S, dishes were washed three times with ice-cold PBS, then quenched with 80:20 HPLC-grade methanol:water (Sigma-Aldrich). Dishes were incubated on dry ice at −80°C for 15 minutes. Plates were scraped, the solution removed, and spun at 2,500 × *g* for 5 minutes at 4°C. The supernatant was removed and stored on ice, then the pellet was washed again in quenching solution and re-spun. Supernatants were combined, dried down under N_2_, and stored at −80°C.

Samples were resuspended in 100 µL HPLC-grade water (Fisher Optima) for analysis on a Thermo Fisher Vanquish Horizon UHPLC coupled to an electrospray ionization source (HESI), part of a hybrid quadrupole-Orbitrap high-resolution mass spectrometer (Q Exactive Orbitrap; Thermo Scientific). Data were collected in full scan negative mode at a resolution of 70 K (13, 55). Chromatography was performed using a 100 mm × 2.1 mm × 1.7 µm BEH C18 column (Acquity) at 30°C. Twenty microliters of the sample was injected via an autosampler at 4°C, and flow rate was 200 µL/min. Solvent A was 97:3 water:methanol with 10 mM tributylamine (TBA) (Sigma-Aldrich) adjusted to a pH of 8.2 using approximately 9 mM acetate (final concentration, Sigma-Aldrich). Solvent B was 100% methanol with no TBA (Sigma-Aldrich). Products were eluted in 95% A/5% B for 2.5 minutes, then a gradient from 95% A/5% B to 5% A/95% B over 14.5 minutes, then held for an additional 2.5 minutes at 5% A/95% B. Finally, the gradient was returned to 95% A/5% B over 0.5 minutes and held for 5 minutes to re-equilibrate the column. MS parameters included: scan in negative mode; scan range = 70–1,000 m/z; automatic gain control = 1e6, spray voltage = 3.0 kV, maximum ion collection time = 40 ms, and capillary temperature = 350°C. Peaks were matched to known standards for identification. Data analysis was performed using the Metabolomics Analysis and Visualization Engine (MAVEN) software (117). Heatmaps were generated using the Multi Experiment Viewer program.

### Kiss and spit heat-killed parasite and conditioned media controls

Low-passage ME49 parasites were lysed from host cells, counted, and heat-killed by incubation at 85°C for 30 minutes. As a negative control, media was heated for the same amount of time. A total of 2 × 10^6^ heat-killed parasites, or an equivalent volume of media, were then added to confluent and quiescent 60 mm dishes of HFFs in triplicate. Conditioned media was taken from heavily infected (MOI 0.75) cells prior to host cell lysis, and media from uninfected paired dishes of host cells served as the negative control. Dishes were incubated for 12 hours at 37°C before their metabolites were extracted and analyzed using the above methodology.

### Kiss and spit cytochalasin D negative control

To ensure that the changes we observed in host metabolism were not attributable to the *T. gondii* remaining in the dish, we performed a control to measure the metabolic contribution of the parasites. A total of 2 × 10^6^ ME49 *T. gondii* parasites were incubated in media with 1.5 μM cytochalasin D at 37°C for 12 hours before pelleting the parasites, washing them to remove the media, and then extracting metabolites. A blank control (media with cytochalasin D but no parasites) was treated identically. Metabolites were quantified using HPLC-MS, and metabolites were identified with known standards. Fold change was calculated with respect to the average of “blank control” abundance, then log base two transformed (log_2_ [Abundance / blank control abundance]), with blue being less abundant and yellow being more abundant. Each column represents one of the six replicates.

### U-13C6-glucose metabolomics

Metabolomic analysis and U-13C6-glucose labeling of HFF cells infected with *T. gondii* was performed as previously described (118). HFFs were grown to deep quiescence in 60 mm dishes in triplicate, then (i) infected with 2 × 10^6^ tachyzoites of *T. gondii* ME49 strain for full infection; (ii) an equal amount of parasite treated with 1.5 µM cytochalasin D for kiss and spit; (iii) mock-infected with an equal volume of media as negative control; and (iv) media plus an equal concentration of cytochalasin D as negative control as well. At 9 HPI, the media was changed to glucose-free RPMI 1640 supplemented to 1 g/L with D-glucose-U-13C6 (Sigma-Aldrich #389374). After 15 and 30 minutes of labeling, dishes were washed 3× with ice-cold PBS, then quenched with 80:20 HPLC-grade methanol:water and incubated on dry ice at −80°C for 15 minutes. Plates were scraped, the solution washed twice, and spun at 2,500 × *g* for 5 minutes at 4°C. Supernatants were combined, dried down under N_2_ gas manifold, and resuspended in 100 µL HPLC-grade water for analysis on a Thermo Fisher Vanquish Horizon UHPLC joined by electrospray ionization (negative mode) to a hybrid quadrupole-Orbitrap high-resolution mass spectrometer (Q Exactive Orbitrap; Thermo Scientific). Chromatography was performed using a 100 mm × 2.1 mm × 1.7 µm BEH C18 column (Acquity) at 30°C. Twenty microliters of the sample was injected via an autosampler at 4°C, and flow rate was 200 µL/minute. Solvent A was 97:3 water:methanol with 9 mM acetate and 10 mM TBA with a pH of 8.2 (Sigma-Aldrich). Solvent B was 100% methanol with no TBA (Sigma-Aldrich). Products were eluted in 95% A/5% B for 2.5 minutes, then a gradient of 95% A/5% B to 5% A/95% B over 14.5 minutes, then held for an additional 2.5 minutes at 5% A/95% B. The gradient was returned to 95% A/5% B over 0.5 minutes and held for 5 minutes to re-equilibrate the column. Data analysis was performed using the MAVEN software. The experiment was performed twice in triplicate. Natural isotope correction was performed using the R code previously published (119).

### Metabolomics with chemical inhibition of cN-II

HFFs were seeded in 60 mm dishes in triplicate and allowed to reach confluency. Thirty-six before the procedure, the DMEM media was changed to metabolomic media. Then, each dish was infected with 2 × 10^6^ ME49 or Pru or PruΔHXGPRT tachyzoites and treated with 50 μM fludarabine or solvent only control. At specific time points, dishes were washed three times with PBS, spun, and then metabolites were extracted and analyzed using the previously mentioned methodology (Fig. 6A). Ten micromolars of AMP, GMP, IMP, adenosine, adenine, guanine, guanosine, and inosine HPLC standard was analyzed simultaneously.

### Fludarabine growth assay

Confluent HFFs were infected with low-passage ME49 and were left to replicate for 4 hours along with uninfected controls. After 4 hours, the media was changed on all cells, creating five different treatment populations: uninfected with DMSO, infected with DMSO, infected with 1 μM pyrimethamine (Sigma-Aldrich), and either 50, 25, or 5 μM fludarabine (Sigma-Aldrich) treatment. Triplicate samples for each of the five conditions were grown for 48 hours, and later 1 μCi of [^3^H] uracil was added. After a 24 hour growth incubation, monolayers were fixed by adding ice-cold 0.6 M trichloroacetic acid (and incubating at 4°C for 1 hour to fix the cells. Trichloroacetic acid was removed, the cells were washed with water for 4 hours. Then 1 M NaOH was added to resolubilize the monolayer, and plates were shaken for 1 hour at room temperature. Each sample was diluted 1:10 in scintillation fluid and [^3^H] abundance was measured. The uninfected population measured baseline host [^3^H] uracil uptake, infected cells treated with DMSO were the control for normal growth, and infected cells treated with pyrimethamine were the control for growth inhibition. Background host [^3^H] incorporation was subtracted from all samples, then the following equation was used to calculate percent inhibition in each sample condition by normalizing to the negative control:


$$Percent\;Growth\;Inhibition=\left(1-\frac{Average\;Sample\;\left[3H\right]\;Count}{DMSO\;Control\;\left[3H\right]\;Count}\right)\times 100$$

### Click chemistry experiments

MDAMB231 parental and MDAMB231 cN-II-KO cells were seeded in 24-well plates with coated glass coverslips. When cells reached 60% confluency, they were infected with 40,000 *T. gondii* tachyzoites of either Pru parental or PruΔHXGPRT parasites. The infection was performed in metabolomic media (RPMI 1640 supplemented with 2 mM L-glutamine, 1% FBS dialyzed against PBS (MW cutoff of 10 kDa), 10 mM HEPES, and 1% penicillin-streptomycin) with 10 µM 7-deaza-7-ethynyl-2′-deoxyinosine (EdI). EdI was synthetized as previously described (120). Twenty micromolar EdI was added to each well of cells. At 48 HPI, cells were gently washed and fixed with 4% paraformaldehyde for 30 minutes at room temperature. Then, cells were permeabilized and blocked with 0.03% Triton X-100 plus 1% bovine serum albumin (AlbuMAX) overnight at 4°C. Followed by PBS gently washes; the cells were incubated with mouse *T. gondii-*chronic serum at 1/500 dilution in 0.03% Triton X-100 plus 1% AlbuMAX for 1 hour at room temperature. After PBS gently washes, cells were incubated in the dark with anti-mouse conjugated serum to Alexa-Azide-588, at 1/500 dilution in 0.03% Triton X-100 plus 1% AlbuMAX for 1 hour at room temperature. After PBS gentle washes, click chemistry reagent (100 mM CuSO_4_, 100 mM sodium L-ascorbate in 0.03% Triton X-100 plus 1% AlbuMAX) was added and incubated in the dark for 1 hour at room temperature. After PBS gentle washes, nuclear staining was performed with DAPI. Imaging was performed in a ZEISS LSM 800 microscope equipped with Zeiss Efficient Navigation (Zen) software. Images were obtained with a 100× oil-immersion objective.

### Nucleotidase activity assay

Nucleotidase activity was evaluated by measuring the inorganic phosphate release upon hydrolysis of IMP or GMP by cN-II enzyme. The Malachite Green Phosphate Assay Kit (BioAssay Systems) is based on the quantification of the green complex formed between Malachite Green, molybdate, and free orthophosphate.

HFF or MDAMB231 cells were seeded in 60 mm dishes using metabolic media and allowed them to reach confluency and quiescence. After that, dishes were treated, infected, or kiss and spit with 4 × 10^6^ parasite tachyzoites per dish for 12 HPI. For treatments, 500 µM 2,3-BPG, or 50 µM Fludarabine by plate were used. Then, plates were washed carefully, and protein lysate was prepared in 1 mL of triton buffer (0.2% Triton X-100, Tris-HCl, 1 mM EDTA, 1 mM DTT, and protease inhibitors, pH 8), thawed at −80°C for 15 minutes, sonicated for 15 seconds, and centrifuged at 2,500 rpm for 5 minutes. The supernatant was used for the following steps. Then, 42 µL of cell lysate in 158 µL of buffer containing (50 mM imidazole pH 6.5, 500 mM NaCl, 10 mM MgCl2, 1 mM DTT) was prepared on ice and the reaction was started by the addition of the substrate (50, 100, or 200 µM of IMP or GMP). Inorganic phosphate was first quantified in the absence of IMP or GMP to evaluate the unspecific hydrolysis and considered as negligible compared to IMP or GMP when used as substrates (data not shown). The volume of the reaction was adapted for 96-well plate according to the manufacturer recommendations by adding 80 µL per well. After 30 minutes at 37°C, the reaction was stopped by addition of 20 µL per well of green malachite reagent on ice, and signal readouts were done 30 minutes later. Free phosphate was quantified by reading the absorbance at 630 nm on a plate reader (Synergy HT). The phosphate concentrations were calculated by a standard curve with phosphate standards. Readouts were normalized to cell lysed protein concentration, which was measured by BCA at 562 nm.

### Statistical analyses

Biological repeats are defined as separate infection time points collected on 2 separate days. GraphPad Prism 10.0.0 was used to create graphs and run statistical analyses. The graphs represent the average of two independent experiments with different replicates. Each statistical analysis test used is described in the legend of each figure. One-way ANOVA or separate *t*-test to compare conditions were used. Tukey’s honestly significant difference method was used to compare the type I error when conducting multiple pairwise comparisons. All reported *P*-values were two-sided, and *P* < 0.05 was used to define statistical significance.

## Data Availability

Authors can confirm that all relevant data are included in the paper and/or its supplementary information files. The LC-MS data generated in this study have been deposited in the metabolomics workbench database DRYAD. The *m/z* cloud database is available, and all the dataset files can be downloaded. Fig. 1; Fig. S1A Part 1: Kiss and spit metabolomics highlight the role of host purine metabolism during pathogen infection https://doi.org/10.5061/dryad.b2rbnzsjd Fig. 2; Fig. S3A Part 2: Kiss and spit metabolomics highlight the role of host purine metabolism during pathogen infection https://doi.org/10.5061/dryad.69p8cz9b5 Fig. 6B; Fig. S5A: Part 3: Kiss and spit metabolomics highlight the role of host purine metabolism during pathogen infection https://doi.org/10.5061/dryad.9p8cz8wrn Fig. 9: Part 4: Kiss and spit metabolomics highlight the role of host purine metabolism during pathogen infection https://doi.org/10.5061/dryad.7d7wm383s Fig. 10A; Fig. S6 Part 5: Kiss and spit metabolomics highlight the role of host purine metabolism during pathogen infection https://doi.org/10.5061/dryad.ghx3ffbxx Fig. S2 Part 6: Kiss and spit metabolomics highlight the role of host purine metabolism during pathogen infection https://doi.org/10.5061/dryad.zkh1893jn Click chemistry quantification code https://github.com/skalalab/Inosine_ClickChemistry.

## SUPPLEMENTAL MATERIAL

Figure S1 msphere.00256-26-s0001.tif Complete *T. gondii* kiss and spit metabolomics.

Figure S2 msphere.00256-26-s0002.tif Kiss and spit controls.

Figure S3 msphere.00256-26-s0003.tif U-13C6 glucose labeling percentage in metabolites in *T. gondii* kiss and spit and full infected HFF cells.

Figure S4 msphere.00256-26-s0004.png Complete conserved shifts in nucleotide metabolism for full infection and kiss and spit.

Figure S5 msphere.00256-26-s0005.tif Effect of CN-II inhibition on *T. gondii* metabolism and replication.

Figure S6 msphere.00256-26-s0006.tif Effect of AMP addition on purine metabolism in *T. gondii*-infected host cells.

Figure S7 msphere.00256-26-s0007.tif Clickable purine analysis to visualize inosine incorporation on infected cells with and without deletion of cN-II enzyme.

Supplemental Legends msphere.00256-26-s0008.docx Supplemental figure and table legends.

Table S1 msphere.00256-26-s0009.docx Host cytosolic nucleotidase enzyme information and comparison.

Table S2 msphere.00256-26-s0010.xlsx Kiss and spit metabolomics.

ASM does not own the copyrights to Supplemental Material that may be linked to, or accessed through, an article. The authors have granted ASM a non-exclusive, world-wide license to publish the Supplemental Material files. Please contact the corresponding author directly for reuse.
