# Supplementary figures and images for: Kiss and spit metabolomics highlight the role of host purine metabolism during pathogen infection
Source: mSphere. 2026 Jun 15;11(7):e00256-26. doi: 10.1128/msphere.00256-26 (PMC13410994; doi:10.1128/msphere.00256-26)

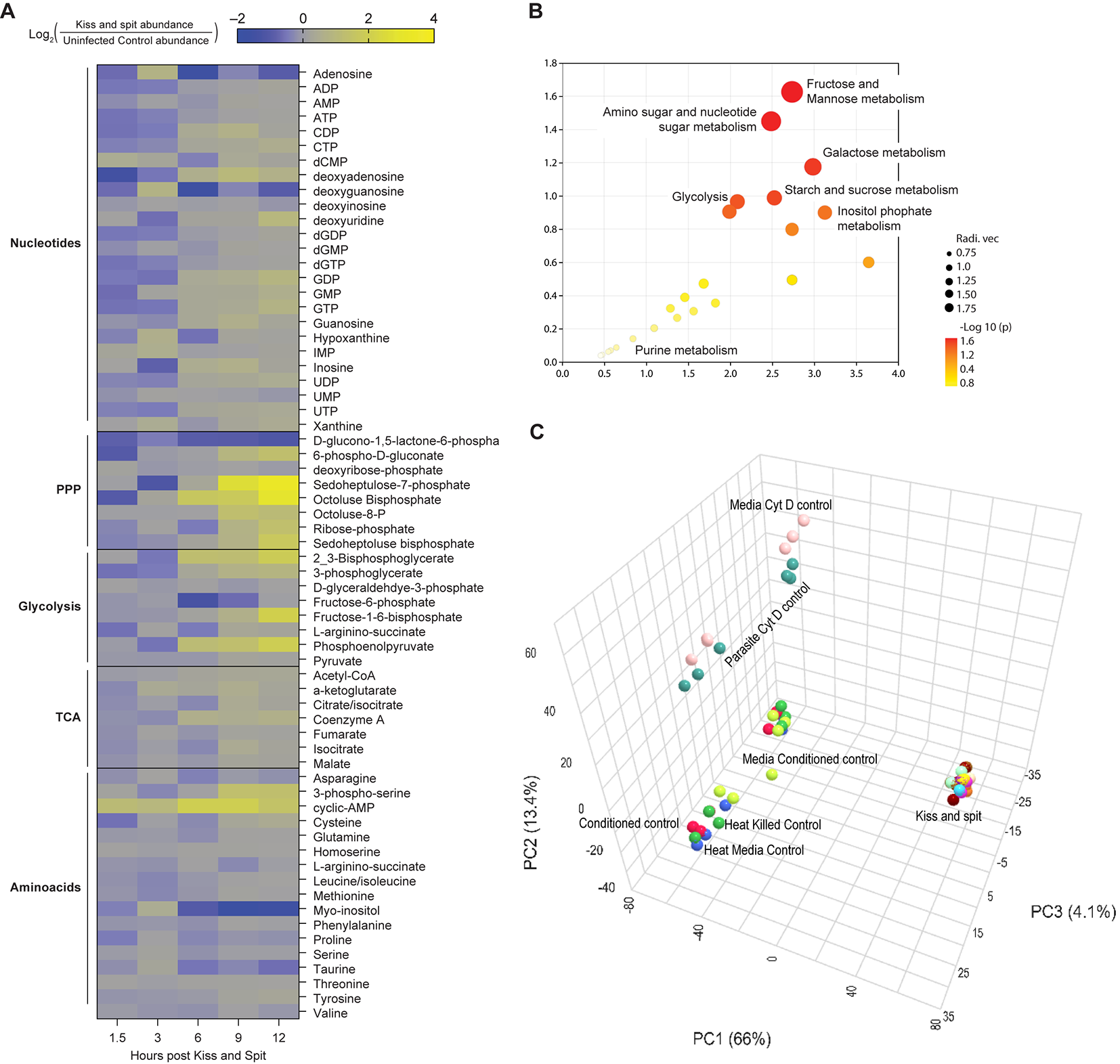

Supplement: Figure S1 — Complete T. gondii kiss and spit metabolomics. [file msphere.00256-26-s0001.tif]

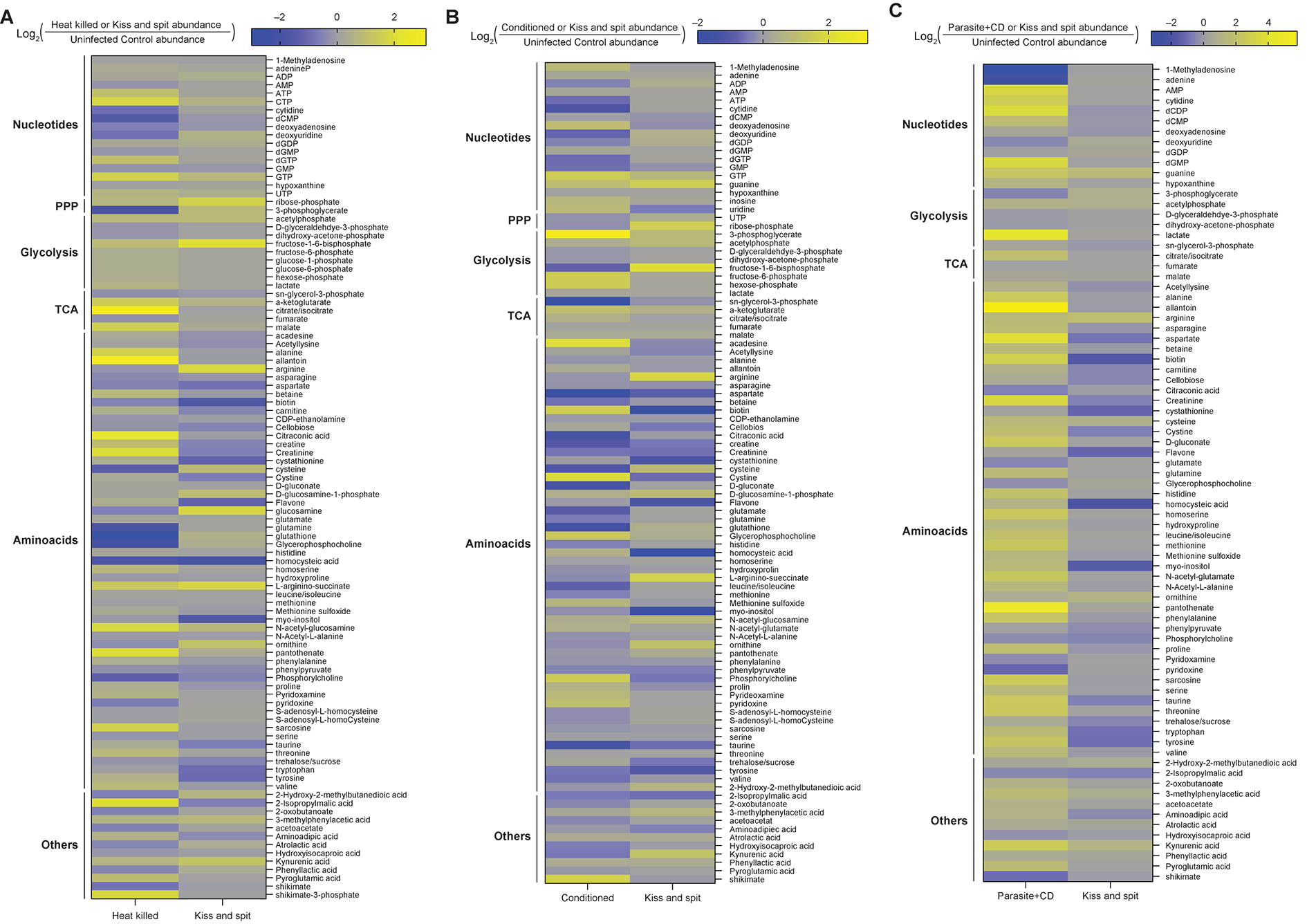

Supplement: Figure S2 — Kiss and spit controls. [file msphere.00256-26-s0002.tif]

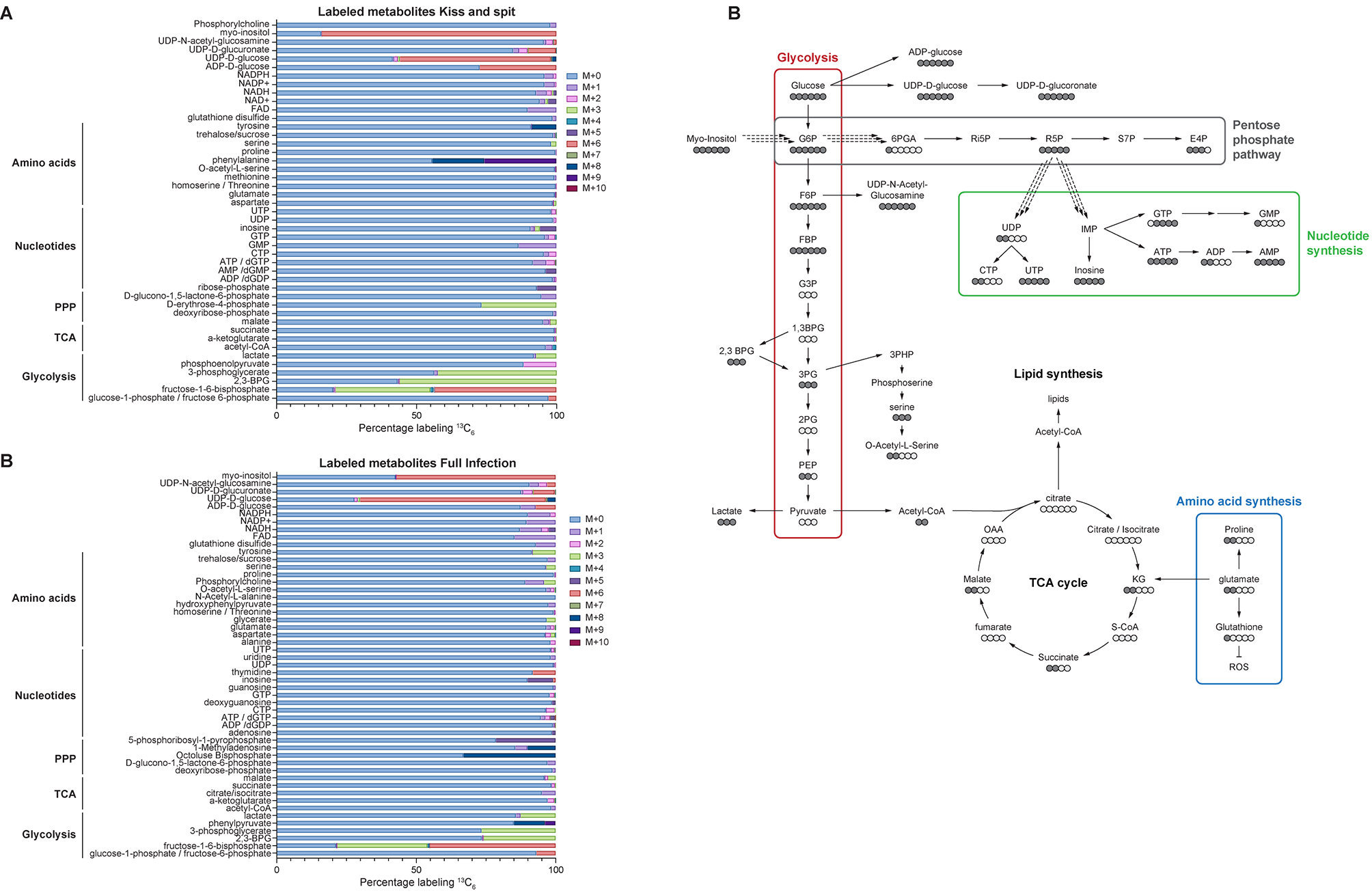

Supplement: Figure S3 — U-13C6 glucose labeling percentage in metabolites in T. gondii kiss and spit and full infected HFF cells. [file msphere.00256-26-s0003.tif]

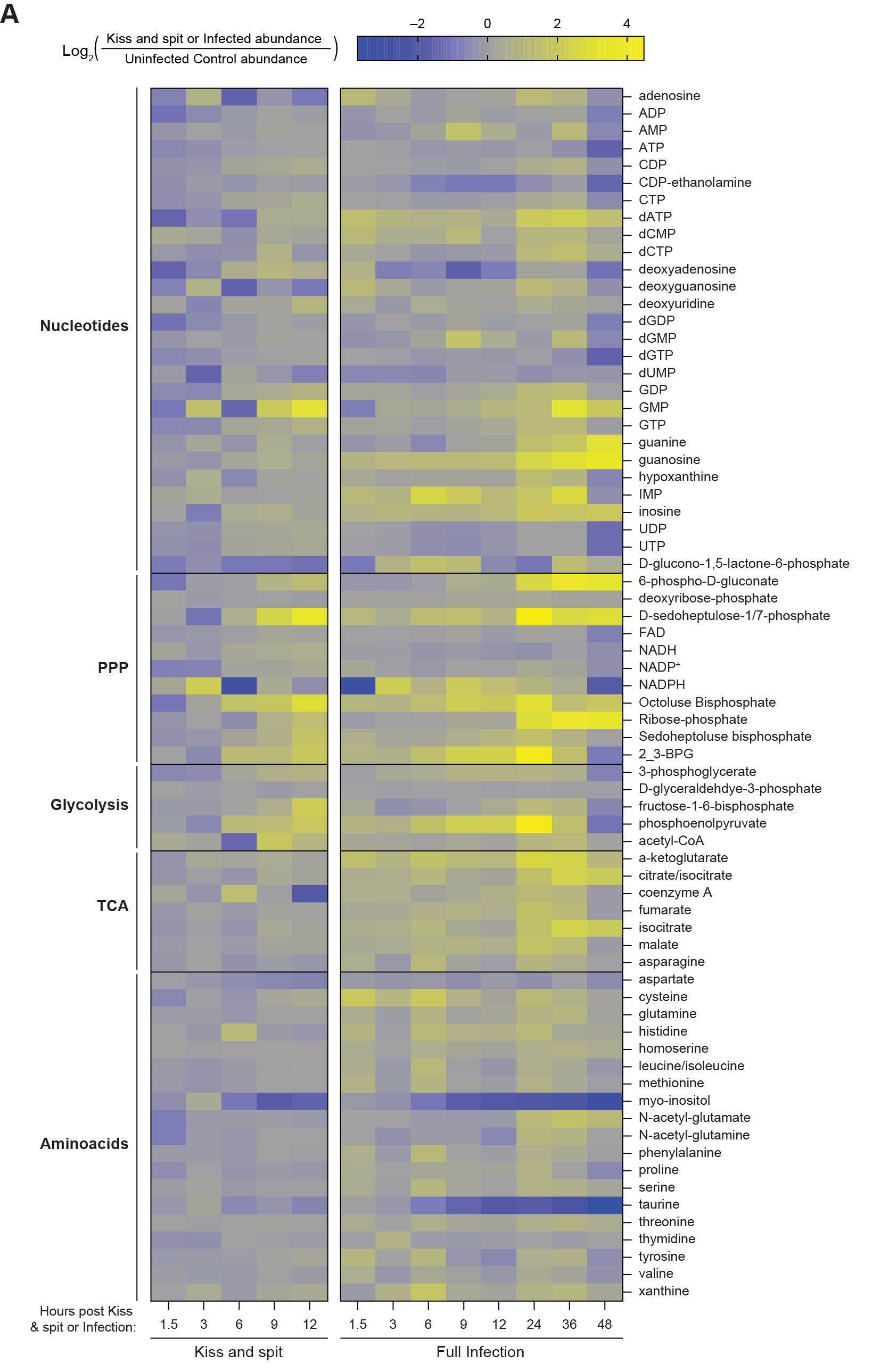

Supplement: Figure S4 — Complete conserved shifts in nucleotide metabolism for full infection and kiss and spit. [file msphere.00256-26-s0004.png]

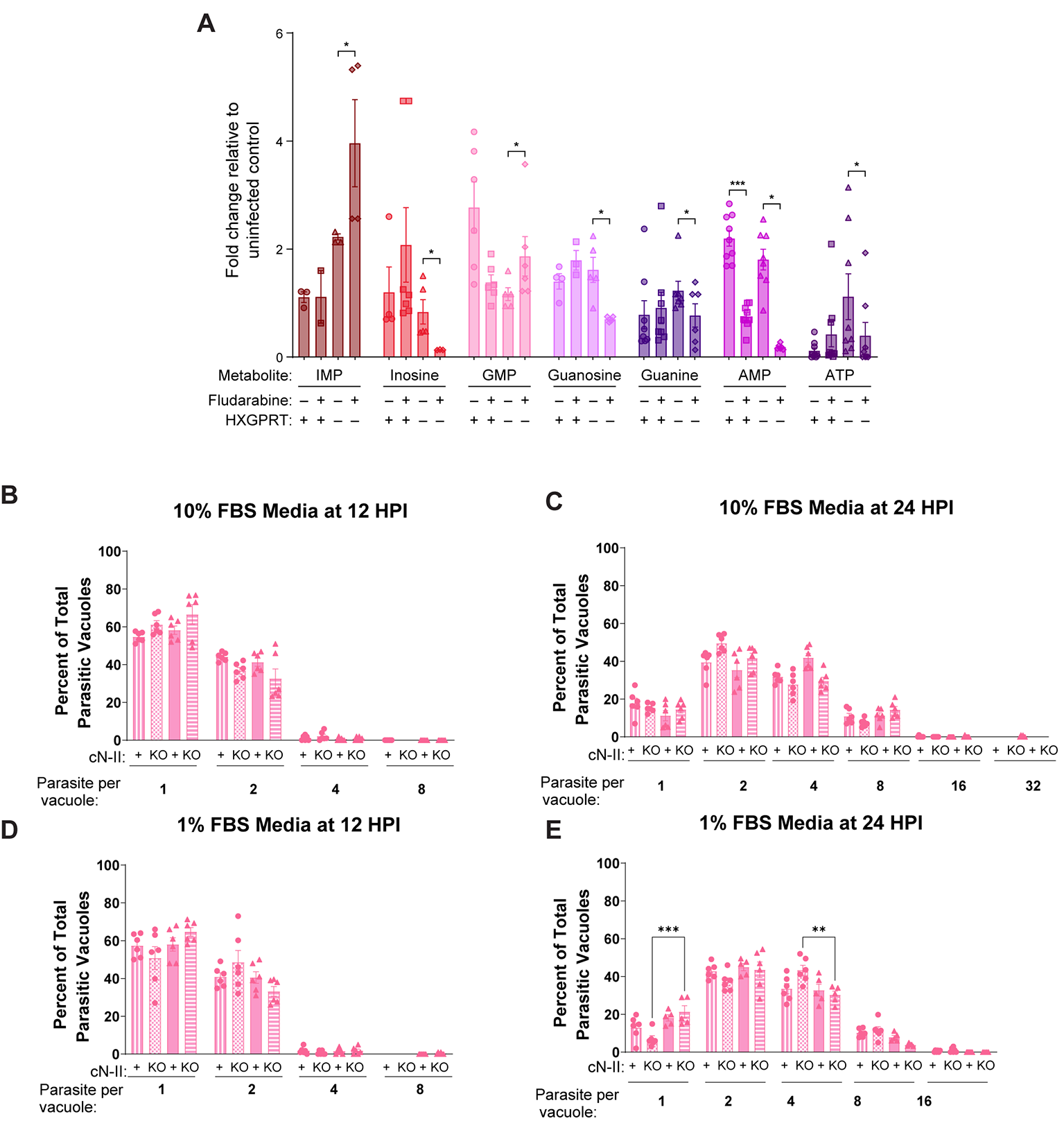

Supplement: Figure S5 — Effect of CN-II inhibition on T. gondii metabolism and replication. [file msphere.00256-26-s0005.tif]

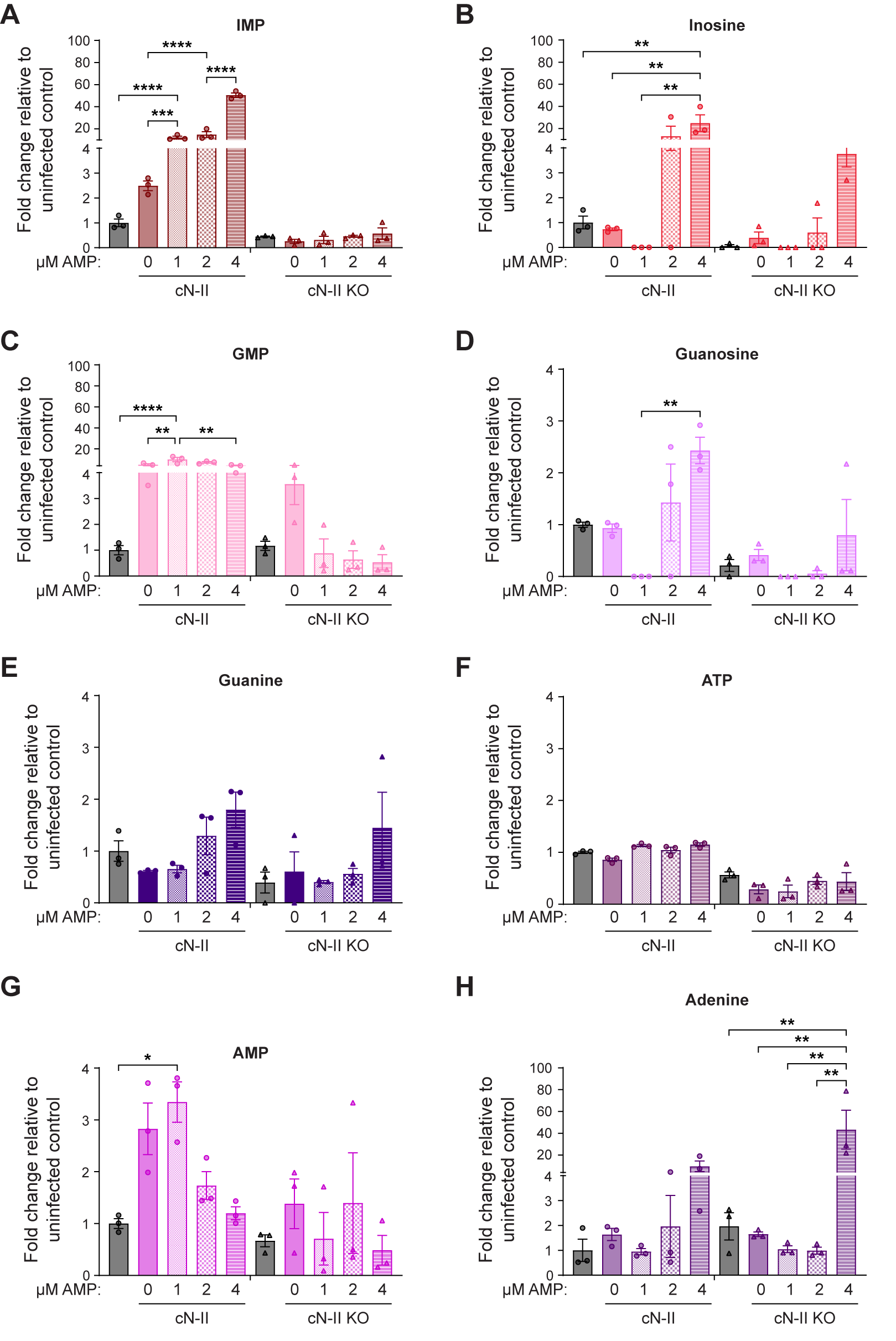

Supplement: Figure S6 — Effect of AMP addition on purine metabolism in T. gondii-infected host cells. [file msphere.00256-26-s0006.tif]

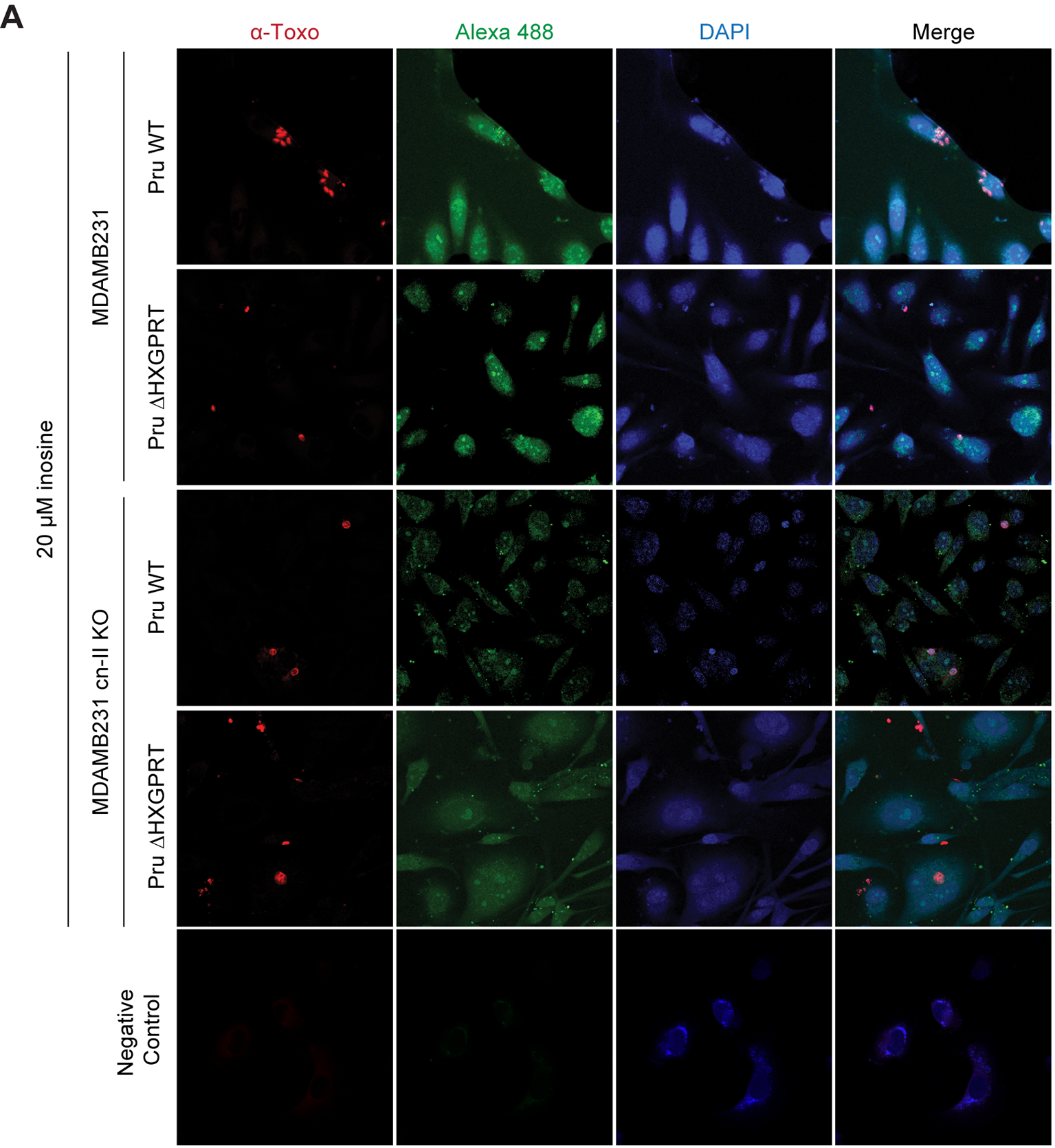

Supplement: Figure S7 — Clickable purine analysis to visualize inosine incorporation on infected cells with and without deletion of cN-II enzyme. [file msphere.00256-26-s0007.tif]
