# Supplementary material for: Kiss and spit metabolomics highlight the role of host purine metabolism during pathogen infection
Source: mSphere. 2026 Jun 15;11(7):e00256-26. doi: 10.1128/msphere.00256-26 (PMC13410994; doi:10.1128/msphere.00256-26)
Supplement: Supplemental Legends — Supplemental figure and table legends. [file msphere.00256-26-s0008.docx]

**Supplementary Figure Legends**

**Supplementary Figure 1**: **Complete *T. gondii* kiss and spit metabolomics**. **(A)** Heatmap shows metabolite relative abundance in human cells over 12 hours after *T.* gondii kiss and spit. Triplicate kiss and spit treated and untreated dishes of HFFs were metabolically quenched (N = 3), and metabolites were extracted at 5 times points (1.5, 3, 6, 9, and 12 HPK&S). Metabolomes were quantified using HPLC-MS and metabolites were identified with known standards. Kiss and spit treated sample abundances were averaged and normalized to the average control abundance then log base 2 transformed (Log_2_ (kiss and spit abundance/Control abundance)) with blue being less abundant and yellow color more abundant. Metabolites were selected for inclusion based on whether they could be confidently identified, and whether their abundance changed during infection. Values represented in this heat map and their *p*-value (calculated by Two-way ANOVA adjust by Tukey’s multiple comparisons) are in Table S2E. **(B)** Global analysis of kiss and spit metabolomics**.** Functional Analysis of predominant metabolic pathways in *T. gondii* kiss and spit. Parameters used: enrichment method: hypergeometric test; topology analysis: relative-betweenness centrality. variance filter: standard deviation; mean intensity value**;** normalization: log transformation, auto-scaling**;** reference metabolome: *Homo sapiens* KEGG**.** Specific values are organized as supplementary tables. Analysis was performed in MetaboAnalyst version 6.0. **(C).** Multivariate comparison of kiss and spit and controls using synchronized 3D plot scatter in MetaboAnalyst version 6.0.

**Supplementary Figure 2: Kiss and spit controls. (A**) heat-killed parasites control. low pass ME49 *T. gondii* parasites were lysed from host cells, counted, and heat-killed by incubation at 85°C for 30 minutes. As a negative control, empty media was also heated for the same amount of time. 2 x 10^6^ heat-killed parasites, or an equivalent volume of empty media was then added to confluent and quiescent dishes of HFFs in triplicate. Dishes were incubated for 12 hours at 37°C before having their metabolites extracted and analyzed. Heatmap shows metabolite abundance in human cells over 12 hours after treatment. Metabolites were quantified using HPLC-MS and identified with known standards. Fold change was calculated with respect to the average of “heat media” control abundance then log base 2 transformed (Log_2_ (abundance/control abundance) with blue being less abundant and yellow color more abundant. Each column represents an average of 6 replicates. Values represented in this heat map and their *p*-value (calculated by multiple t-test) are in Table S2F. **(B).** Kiss and spit conditioned media control. Conditioned media was taken from heavily infected cells prior to host cell lysis and the release of parasites into the media. Media from uninfected paired dishes of host cells served as the negative control. Confluent and quiescent dishes of HFFs were incubated in each media condition for 12 hours at 37°C before having their metabolites extracted and analyzed. Heatmap shows metabolite abundance in human cells over 12 hours after treatment. Metabolites were quantified using HPLC-MS and identified with known standards. Fold change was calculated with respect to the average of “uninfected media control” abundance then log base 2 transformed (Log_2_ (abundance/control abundance) with blue being less abundant and yellow color more abundant. Each column represents an average of 6 replicates. Values represented in this heat map and their *p*-value (calculated by multiple t-test) are in Table S2F. **(C).** Kiss and spit - cytochalasin D negative control. 2x10^6^ parasites were incubated in media with 1.5 μM cytochalasin D at 37°C for 12 hours before pelleting the parasites, washing them to remove the media, and then extracting metabolites. A blank control (media with cytochalasin D but no parasites) was treated identically. Heatmap shows metabolite abundance samples over 12 hours after treatment. Metabolites were quantified using HPLC-MS and identified with known standards. Fold change was calculated with respect to the average of “blank control” abundance then log base 2 transformed (Log_2_ (abundance/blank control abundance) with blue being less abundant and yellow color more abundant. Each column represents an average of 6 replicates. Values represented in this heat map and their *p*-value (calculated by multiple t-test) are in Table S2F.

**Supplementary Figure 3:** **U-13C6 Glucose labeling percentage in metabolites in *T. gondii* kiss and spit and full infected HFF cells.** U-13C6 glucose labeling was performed for 30 minutes after 9 HPI in ME49 *T. gondii* kiss and spit and full infected HFF cells. Metabolites were quantified using HPLC-MS and metabolites were identified with known standards, data was corrected for natural abundance. N = 6. **(A)** Kiss and spit or **(B)** full infection percentage of labeling is indicated on the x axis which represents the mean of 7 replicates. Metabolites shown on the Y axis, were selected for inclusion based on whether they could be confidently identified, and their labeling was constant in kiss and spit and full infection. ‘M + n’ (‘M’ stands for molecular mass; n indicates the number of heavy carbons ^13^C in the metabolite). Each color represents a different labeling from M+0 to M+8. Values represented in this heat map and their *p*-value (calculated by two-way ANOVA with Bonferroni’s multiple comparison test) are in Table S2G. **(C)** U-13C6 label integration into pathways of the central carbon metabolism during *T. gondii* kiss and spit. Pictogram of glycolysis, TCA cycle, pentose phosphate pathway, as well as purine, pyrimidine and amino acids biosynthesis. Some metabolites and metabolic routes were not represented or simplified for better visualization. Gray circles represent glucose-derived 13C atoms. Unlabeled C atoms are displayed in white. This figure was build based on our results and literature (60–74).

**Supplementary Figure 4. Complete conserved shifts in nucleotide metabolism for full Infection and kiss and spit.** Heat map of metabolites that are significantly up (yellow) or down (blue) regulated in response to ME49 *T. gondii* kiss and spit (left panel) or full infection (right panel). Hours post-infection is across the bottom of the heat map, 1.5, 3, 6, 9, 12, 24, 36, or 48. N = 3 for each time point. Values represented in this heat map and their *p*-value (calculated by multiple t-test) are in Table S2H

**Supplementary Figure 5: Effect of CN-II inhibition on *T. gondii* metabolism and replication.**  **(A)** Effect of fludarabine on purine metabolism in *T. gondii* in host cells. Relative abundance of selected purine metabolites in HFF cell line infected with Pru WT or PruΔHXGPRT *T. gondii* and treated with fludarabine at 24 HPI. Each bar represents the abundance mean of two independent experiments (N = 4-9) normalized to the abundance mean of the uninfected control. Statistical analyses were performed using multiple t-tests. 0.1234 (ns), 0.0332 (*), 0.0021 (**), 0.0002 (***), <0.0001(****). Pru WT infected HFF are represented by circle symbols, Pru WT infected + fludarabine are represented in square symbols, PruΔHXGPRT infected HFF cells are represented in triangle symbols and PruΔHXGPRT infected HFF cells + fludarabine are represented in rhombus symbols. Pru WT *T. gondii* is represented with HXGPRT +, PruΔHXGPRT *T. gondii* is represented by HXGPRT - and treatment with fludarabine is represented with +. **(B)** Effect of cN-II gene deletion in *T. gondii* replication in infected cells. Replication of the parasite was evaluated by counting the parasites per vacuole at 12 and 24 HPI in Pru WT and Pru-ΔHXGPRT *T. gondii* infected MDAMB231 cells with and without cN-II knock-out by two expert scientists. The number of parasites per vacuole is shown in the X axis and the percentage of total parasite vacuoles is shown in the Y axis. **(B)** intracellular parasites grew in media with 10% FBS during 12 HPI; **(C)** intracellular parasites grew in media with 10% FBS during 24 HPI. **(D)** intracellular parasites grew in media with 1% FBS during 12 HPI; **(E)** intracellular parasites grew in media with 1% FBS during 24 HPI. Bar graphs show the average of the six replicates and the error bar shows the SEM. Statistical analyses were performed by 2-way ANOVA. 0.1234 (ns), 0.0332 (*), 0.0021 (**), 0.0002 (***), <0.0001(****). Infected MDAMB231 cells are represented with (+) and infected MDAMB231 cN-II KO cells are represented with KO Pru WT parasites are represented by circular symbols and Pru-ΔHXGPRT represented by triangle symbols. Each dot represents an independent replicate, the graph bar shows the average of 6 replicates, and the error bars represent the SEM.

**Supplementary Figure 6:** **Effect of AMP addition on purine metabolism in *T. gondii* infected host cells.** Relative abundance of selected purine metabolites in MDAMB231 cell line with and without cN-II genetic deletion and infected with ME49 *T. gondii* and increasing addition of AMP (0, 1, 2, 4 µM): **(A)** IMP**.** **(B)** Inosine. **(C)** GMP**.** **(D)** Guanosine. **(E)** Guanine**.** **(F)** ATP. **(G)** AMP**.** **(H)** Adenine. Metabolites were quantified using HPLC-MS and identified with known standards. Each graph bar represents the mean fold change of selected metabolites in infected cells with respect to the uninfected control in triplicate. Each graph bar represents the mean of 3 replicates and error bars represent the SEM. Statistical analysis was performed by one- way ANOVA with Tukey’s multiple comparisons to compare different supplementation of AMP in MDAMB231 or MDAMB231 cN-II KO cells. Alpha = 0.05. 0.1234 (ns), 0.0332 (*), 0.0021 (**), 0.0002 (***), <0.0001(****). Infected MDAMB231 cells are represented with circular symbols and infected MDAMB231 cN-II KO cells are represented with triangle symbols. Values represented in this figure and *p*-values are in Table S2I

**Supplementary Figure 7.** **Clickable purine analysis to visualize inosine incorporation on infected cells with and without deletion of cN-II enzyme*.*** **(A)**. Inosine (EdI) incorporation and Cu-catalyzed azide-alkyne staining of active intracellular parasites in MDA-MB231 parental, and MDAMB231 cN-II KO host cells infected with Pru WT or PruΔHXGPRT *T. gondii* parasites at 48 HPI. EdI was added to 20 µM in cell culture media. α-*Toxoplasma* localizes in red color the *T. gondii* parasite detected with chronic *T. gondii* infected mice serum. Alexa 488 localizes in green color the incorporation of EdI. DAPI staining localizes in blue color the cell and parasite nuclei. Merged localized the morphology of cells and parasites. The representative images displayed in this figure were increased in resolution for better visualization.

**Supplementary Tables**

**Supplementary Table 1: host cytosolic nucleotidase enzymes information and comparison.**

**Supplementary Table 2: kiss and spit metabolomics. (A)**This table contains the exact values for the ratios represented in Fig. 1B. heat maps and their statistical analysis with *p*-values included**. (B)** U-13C6 labeling in *T. gondii* infection kiss and spit and full infection. This table contains the exact values for the ratios represented in Fig. 2 and their P Value for statistical analysis. **(C)** Comparison of selected conserved shifts in nucleotide metabolism for full Infection and kiss and spit. This table contains the exact values for the ratios represented in Fig. 3A and their statistical analysis with *p*-values included. **(D)** AMP supplementation effect on purine metabolism in *T. gondii* infection. This table contains the exact values and statistical analysis for Fig. 10A. **(E)**This table contains the exact values for the ratios represented in Fig. S1. heat maps and their statistical analysis with *p*-values included **(F)** controls kiss and spit metabolomics. This table contains the exact values for the ratios represented in Figs. S2 heatmaps, and their statistical analysis with *p*-values included**. (G)** U-13C6 labeling in *T. gondii* infection kiss and spit and full infection. This table contains the exact values for the ratios represented in Fig. S3 and their P Value for statistical analysis. **(H)** Comparison of selected conserved shifts in nucleotide metabolism for full Infection and kiss and spit. This table contains the exact values for the ratios represented in Fig S4 and their statistical analysis with *p*-values included. **(I)**. AMP supplementation effect on purine metabolism in *T. gondii* infection. This table contains the exact values and statistical analysis for Fig. S6**. (J)** Primer sequences. This table contains the exact sequence of primers used for QPCR shown in Figs. 5B and Fig. 7.
