# Supplementary material for: Kiss and spit metabolomics highlight the role of host purine metabolism during pathogen infection
Source: mSphere. 2026 Jun 15;11(7):e00256-26. doi: 10.1128/msphere.00256-26 (PMC13410994; doi:10.1128/msphere.00256-26)
Supplement: Table S1 — Host cytosolic nucleotidase enzyme information and comparison. [file msphere.00256-26-s0009.docx]

| Enzyme | Names | Alias | Expression | Function | Diseases | NCBI | Official symbol / Reference sequence | Gene ID | Cellular localization | References |
| --- | --- | --- | --- | --- | --- | --- | --- | --- | --- | --- |
| cN1 A | Cytosolic 5'-nucleotidase IA, NT5C1A | AMP-specific 5'-NT; cN-I | High expression in heart, brain, prostate, Testis | Act on AMP, IMP, GMP, XMP, dAMP, dGMP. | Breast Cancer, Pancreatic cancer, Autoimmune diseases | <https://www.ncbi.nlm.nih.gov/gene/84618> | NT5C1A | 84618 | Cytosol | ^1^,^2^ |
| cN1 B | Cytosolic 5'-nucleotidase IB, NT5C1B | cN-IA homolog; AIRP | High expression in Testis | Production of Adenosine, act on XMP | Autoimmune diseases | <https://www.ncbi.nlm.nih.gov/gene/93034> | NT5C1B | 93034 | Cytosol, nucleus | ^1^,^2^ |
| cN II | Cytosolic 5'-nucleotidase II, NT5C2 | High Km 5'-NT; purine 5'-NT; GMP, IMP-specific 5'-NT | Have 30 variants, ubiquitous, thyroid, esophagus | Hydrolase acting on IMP, GMP | Osteoarthritis, Leukemia, colon cancer, heart disease, autism spectrum disorder | [NT5C2 5'-nucleotidase, cytosolic II [Homo sapiens (human)] - Gene - NCBI (nih.gov)](https://www.ncbi.nlm.nih.gov/gene/22978) | NM_012229.5 | 22978 | Cytosol | ^3^, ^2^, ^4^,^5^,^6^, ^7^ |
| cN 3A | Cytosolic 5'-nucleotidase III, NT5C3 | PN-I; P5'N-1; UMPH, P36 | High expression in Red Blood Cells, Colon, duodenum, various organs | Degradation RNA, Pyrimidine nucleotidase (CMP preferred substrate, also UMP). Inactive with purine substrates. Isoform B, able to hydrolyze deoxy pyrimidine nucleotide monophosphate (dCMP); | Lupus and hemolytic anemia | <https://www.ncbi.nlm.nih.gov/gene/51251> | NT5C3A | 51251 | Cytosol, nucleoplasm, endoplamic reticulum | ^3^, ^2^, ^8^ |
| cN 3B | Cytosolic 5'-nucleotidase IIIB | 5'-nucleotidase cytosolic III-like, 7-methylguanosine nucleosidase, N(7)-methyl guanylate 5'-phosphatase, cN-III-like protein, cytosolic 5'-nucleotidase 3B, cytosolic 5'-nucleotidase III-like protein | Testis, thyroid, and other tissues | m7GMP. | Lynch syndrome, airway wall thickening and airway obstruction | [NT5C3B 5'-nucleotidase, cytosolic IIIB [Homo sapiens (human)] - Gene - NCBI (nih.gov)](https://www.ncbi.nlm.nih.gov/gene/115024) | NT5C3B | 115024 | Cytosol | ^9^ |
| e-N | Ecto 5- Nucleotidase | NT5E, Ecto-5’, 5’NT; eNT, CD73 | eN is variably expressed in a wide number of cell types under physiological and pathological conditions, endometrium, oary | Act on AMP, co-receptor in T cell activation, Hydrolyze ATP to adenosine. | Gliobastima, cancer, liver fibrosis, Rheumatoid arthritis | [NT5E 5'-nucleotidase ecto [Homo sapiens (human)] - Gene - NCBI (nih.gov)](https://www.ncbi.nlm.nih.gov/gene/4907) | NT5E | 4907 | Protein bound to the outer surface of the plasma membrane (GPI), Lipid rafts | ^10^,^2^, ^11^ |
| cDN | Cytosolic 5’(3’)-deoxynucleotidase, NT5C | dNT-1, PN-II | Ubiquitous, colon, spleen, | Act on dIMP, dGMP, | Myeloid Leukemia, Pyrimidine deficiency | [NT5C 5', 3'-nucleotidase, cytosolic [Homo sapiens (human)] - Gene - NCBI (nih.gov)](https://www.ncbi.nlm.nih.gov/gene/30833) | NT5C | 30833 | Nucleus, extracellular exosome and cytosol | ^12^,^13^, ^2^ |
| mDN | Mitochondrial 5’(3’)-deoxynucleotidase, NT5M | NT5M, dNT-2 | Testis, bone marrow, | Act on dUMP, dTMP, | Genetic diseases, | [NT5M 5',3'-nucleotidase, mitochondrial [Homo sapiens (human)] - Gene - NCBI (nih.gov)](https://www.ncbi.nlm.nih.gov/gene/56953) | NT5M | 56953 | Mitochondrial matrix | ^14^, ^2^, ^15^ |

**Supplementary Table 1: host cytosolic nucleotidase enzymes information and comparison.**

1. Sala-Newby, G. B., Freeman, N. V. E., Skladanowski, A. C. & Newby, A. C. Distinct roles for recombinant cytosolic 5’-nucleotidase-I and -II in AMP and IMP catabolism in COS-7 and H9c2 rat myoblast cell lines. *The Journal of biological chemistry* **275**, 11666–71 (2000).

2. Bianchi, V. & Spychala, J. Mammalian 5′-Nucleotidases. *Journal of Biological Chemistry* **278**, 46195–46198 (2003).

3. Oka, J., Matsumoto, A., Hosokawa, Y. & Inoue, S. Molecular Cloning of Human Cytosolic Purine 5′-Nucleotidase. *Biochemical and Biophysical Research Communications* **205**, 917–922 (1994).

4. Barsotti, C., Pesi, R., Giannecchini, M. & Ipata, P. L. Evidence for the involvement of cytosolic 5’-nucleotidase (cN-II) in the synthesis of guanine nucleotides from xanthosine. *The Journal of biological chemistry* **280**, 13465–9 (2005).

5. Pesi, R. *et al.* The bifunctional cytosolic 5′-nucleotidase: Regulation of the phosphotransferase and nucleotidase activities. *Archives of Biochemistry and Biophysics* vol. 312 75–80 Preprint at https://doi.org/10.1006/abbi.1994.1282 (1994).

6. Pesi, R. *et al.* Cytosolic 5’-Nucleotidase II Silencing in a Human Lung Carcinoma Cell Line Opposes Cancer Phenotype with a Concomitant Increase in p53 Phosphorylation. *International journal of molecular sciences* **19**, 1–20 (2018).

7. Ipata, P. L. & Tozzi, M. G. Recent advances in structure and function of cytosolic IMP-GMP specific 5’-nucleotidase II (cN-II). *Purinergic signalling* **2**, 669–75 (2006).

8. Rich, S. A., Bose, M., Tempst, P. & Rudofsky, U. H. Purification, microsequencing, and immunolocalization of p36, a new interferon-alpha-induced protein that is associated with human lupus inclusions. *The Journal of biological chemistry* **271**, 1118–26 (1996).

9. Monecke, T., Buschmann, J., Neumann, P., Wahle, E. & Ficner, R. Crystal structures of the novel cytosolic 5′-nucleotidase IIIB explain its preference for m7GMP. *PLoS ONE* **9**, 1–13 (2014).

10. MISUMI, Y., OGATA, S., OHKUBO, K., HIROSE, S. & IKEHARA, Y. Primary structure of human placental 5′‐nucleotidase and identification of the glycolipid anchor in the mature form. *European Journal of Biochemistry* **191**, 563–569 (1990).

11. Fausther, M. *et al.* Coexpression of ecto-5′-nucleotidase/CD73 with specific NTPDases differentially regulates adenosine formation in the rat liver. *American Journal of Physiology-Gastrointestinal and Liver Physiology* **302**, G447–G459 (2012).

12. Rampazzo, C. *et al.* Mammalian 5’(3’)-deoxyribonucleotidase, cDNA cloning, and overexpression of the enzyme in Escherichia coli and mammalian cells. *The Journal of biological chemistry* **275**, 5409–15 (2000).

13. Mazzon, C. *et al.* Cytosolic and mitochondrial deoxyribonucleotidases: activity with substrate analogs, inhibitors and implications for therapy. *Biochemical pharmacology* **66**, 471–9 (2003).

14. Rampazzo, C. *et al.* A deoxyribonucleotidase in mitochondria: Involvement in regulation of dNTP pools and possible link to genetic disease. *Proceedings of the National Academy of Sciences of the United States of America* **97**, 8239–8244 (2000).

15. Gallinaro, L. *et al.* Human mitochondrial 5’-deoxyribonucleotidase. Overproduction in cultured cells and functional aspects. *The Journal of biological chemistry* **277**, 35080–7 (2002).
